# Exploring the effects of increasing underutilized crops on consumers’ diets: the case of millet in Uganda

**DOI:** 10.1186/s40100-021-00206-3

**Published:** 2022-01-05

**Authors:** Cesar Revoredo-Giha, Luiza Toma, Faical Akaichi, Ian Dawson

**Affiliations:** 1 Rural Economy, Environment and Society Department, Scotland’s Rural College (SRUC), King’s Buildings, West Mains Road, Edinburgh, EH9 3JG UK; 2 Knowledge and Innovation Hub, Scotland’s Rural College (SRUC), Edinburgh, UK

**Keywords:** Underutilized crops, Millet, Consumption, Generalized rationing theory, Sub-Saharan Africa, Uganda

## Abstract

Known in the literature as underutilized, neglected or orphan crops, these crops have been cited as having the potential to improve food and nutritional security. The literature also highlights however that consumers in developing countries are increasingly abandoning their traditional diets that these crops are part of, and are replacing them by western diets. In this context, the purpose of this paper is to investigate the consumption and nutritional implications of expanding the participation of underutilized crops in current diets. This was done using a modified version of the microeconomic consumer problem. This was augmented with a linear constraint using generalized rationing theory that can be found in the economics literature. The method was applied to the case study of the consumption of millet (finger millet, botanical name: *Eleusine coracana*) by rural, urban-poor and urban-affluent Ugandan socioeconomic groups. The results indicated that millet could contribute to improving the intake of macronutrients and of some micronutrients, though the overall picture is complex. However, under current preferences and given its demand inelasticity, to achieve a substantial increase in the quantity of millet in the diet will require a significant reduction of its price. Otherwise, the net impact on nutrition as measured by the mean adequacy ratio will be only slightly positive for rural and urban-poor households. Our findings indicate that supply-side initiatives aimed at increasing the productivity of underutilized crops (reducing crop price) are likely to produce disappointing results in restoring their importance unless accompanied by specific interventions to expand demand.

**Supplementary Information:**

The online version contains supplementary material available at 10.1186/s40100-021-00206-3.

## Introduction

Green Revolution research focused on optimising the yields of a few staple crops to support the production of sufficient affordable calories for humans (McMullin et al. 2021). This, however, occurred at the expense of research into the yield, quality improvement and resilience of so-called underutilized, neglected or orphan crops, which provide a better supply of particular nutrients such as essential amino acids, minerals and fibre (FAO 2021).

Some of these underutilized crops have the capacity to be used in the management/feeding of farmed animals, food processing and the wider food system (e.g., Qaim 1999; Dawson et al. 2009; ATDF 2009). Additionally, they can be produced in more sustainable ways than major staple crops, especially when considering the external costs of production to the environment (Dawson et al. 2019; AOCC 2021).

The current strategy on underutilized crops as implemented, for instance, by the African Orphan Crops Consortium (AOCC 2021) focuses on their genetic improvement to improve their productivity and quality and increase their resilience to climate change. This is based on the assumption that these efforts will translate into higher crop production, and then consumption, diversity. However, Sibhatu et al. (2015) found that in developing countries greater production diversity does not necessarily correspond with greater dietary diversity. The connection between production and consumption may be most tenuous in urban areas, due to the adoption of western diets (e.g., Hawkes 2006; Moodie et al. 2013) that displace traditional ones (e.g., Worku et al. 2017). The increased power of multinational food companies, government subsidy patterns that support major staple crop production, farm mechanisation, the consolidation of plant breeding companies, and limited investments in breeding programmes for underutilized crops, have all had a role to play in changing dietary trends toward more staple crop consumption (Khoury et al. 2014).

Action on the consumption of underutilized crops or products derived from them (the demand side) is important as well as production interventions. It helps ensure producers a fair and sustainable return for their products by connecting them with markets, which has been shown to be an effective tool against poverty (African Development Bank Group 2016). In addition, responding to consumer preferences can be seen as a useful approach to support healthy diets in situations where consumers face complex choices. This is needed in Africa for example as consumer markets expand with ultra-processed products (Moodie et al. 2013). These products are displacing more traditional dietary elements of a range of fresh and perishable whole or minimally processed foods, a fact that could be associated with increasing levels of non-communicable diseases (Global Nutrition Report 2018).

The purpose of this paper is to study two aspects of the consumption of underutilized crops: first, the implications of expanding their presence in the diet by considering consumers’ current preferences through demand elasticities; and second, to measure the resulting changes on consumption and nutrition for the entire resulting diet (i.e., not just that impact coming solely from the additional crop). This was done using a version of the microeconomic consumer problem (Jackson 1991) modified by augmentation with a linear constraint (Irz et al. 2015) that enforces a minimum requirement of the underutilized crop in the diet. An interesting methodological output of the work is the computation of the shadow price associated with the underutilized crop constraint. This allows us to understand how much the price of the crop needs to be reduced in order to increase its presence substantially in the diet. If the crop price needs to decrease significantly (comparing the original price with the shadow price), this would indicate that major supply-side measures will be needed to significantly increase crop productivity. In this context, investing in ways to expand demand may achieve greater impact than supply side interventions.

Another contribution of the paper is to measure the share of the underutilized crop in the diet. Most support to try and increase underutilized crop consumption has come from “supply side” researchers (e.g., Dawson et al. 2019; Mayes et al. 2011; Cheng et al. 2017) focusing on improving the characteristics and consumption of individual crops in isolation. But increasing the consumption of any one underutilized crop will have broader implications for the composition of diets and nutrient intake broadly. This could happen, for example, through the relationship with other food products and meeting a budget constraint. Therefore, an evaluation of the nutritional advantages of orphan crops must be done in the context of the diet as a whole.

Here, the above methods are applied to the case study of the consumption of millet in Uganda. Most of the millet planted in Uganda is finger millet, *Eleusine coracana*, but for simplicity we use the shorter term ‘millet’ in this paper. We focus on the crop because it was identified as a priority for research by the African Orphan Crops Consortium (AOCC 2021). In addition, cereals including millet contribute over 40% to total direct human dietary calorie intake in Eastern Africa (Gierend and Orr 2015). Millet also is one among the mandate crops of ICRISAT who promote its production and consumption in this region (Gierend and Orr 2015; Orr et al. 2020).

The selection of Uganda was due to the contraction of the apparent consumption of millet over the last decades, which has been 4.7% per year on average since 1968 according to FAOSTAT figures.^1^ The Government of Uganda is nevertheless interested to expand the crop’s production and consumption. This is illustrated by work at the Mukono Zonal Agricultural Research Institute (Muzardi) that research millet varieties originating from China as part of a Uganda–China partnership (GlobalFoodMate 2013). In addition, millet is one of the cereals considered in Uganda’s National Grain Policy (Uganda Ministry of Trade, Industry and Cooperatives 2015).

Our paper starts with a review of the consumption of millet in the Sub-Saharan Africa region, and then briefly describes broader food consumption patterns in Uganda. This is followed by a presentation of the methods and data used to evaluate the implications of increasing the consumption of millet in Uganda, a discussion of our findings, and our conclusions.

## Consumption of millet in Sub-Saharan Africa

**Fig. 1:**
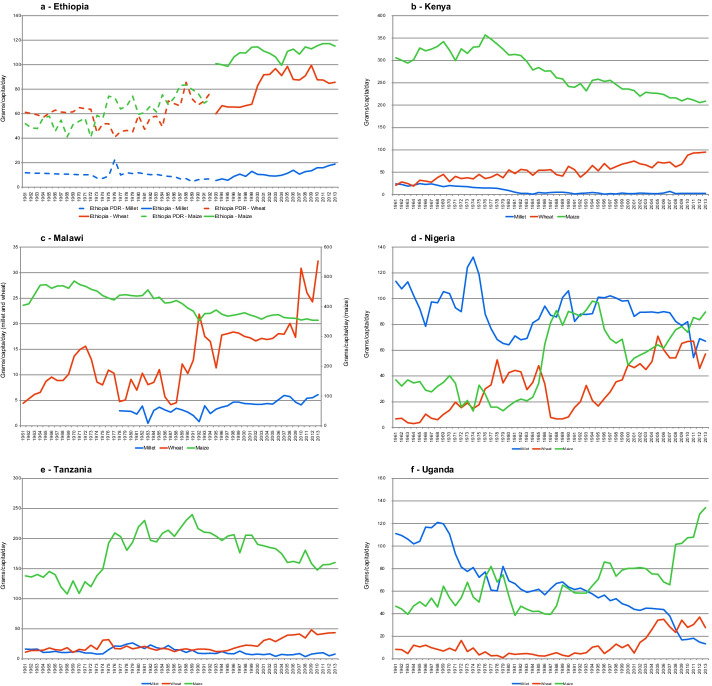
Apparent per capita per day consumption of millet, maize and wheat and their products in selected Sub Saharan African countries. *Source*: Based on FAOSTAT data

Figure 1 shows the apparent per capita consumption of millet, maize and wheat for the time series 1961–2013 in the six Sub-Saharan countries of Ethiopia, Kenya, Malawi, Nigeria, Tanzania and Uganda (a recent review on why to invest in research and development for sorghum and millets in East and Southern Africa can be found in Orr et al. 2020).

Despite its role in African diets, with the exception of Ethiopia and Malawi, countries show decreasing trends in consumption. Moreover, with the exception of Nigeria, much less millet is now (to 2013) consumed than maize and wheat.

The number of studies analysing the actual consumption of millet in Sub-Saharan Africa is limited. Gierend and Orr (2015) focussed on the demand for millet and sorghum in Ethiopia, Kenya, Tanzania and Uganda. They found that while the per capita consumption overall was static, there were differences between countries. In Kenya and Tanzania, consumption per capita did not change between 2000 and 2013; in Ethiopia, annual consumption rose from 4.5 to 8.0 kg/capita; and in Uganda consumption fell from 29 to 5 kg/capita.

Gierend and Orr (2015) also found that, across countries, consumption averaged 7.2 kg/capita in rural areas and 3.7 kg/capita in urban ones. The rural bias was strongest in Ethiopia, at 10.6 and 3.3 kg/capita for rural and urban areas, respectively. But urban demand remained considerable in each country, in absolute terms amounting to 46,000 tonnes in Tanzania, 43,000 tonnes in Uganda, 42,000 tonnes in Ethiopia, and 21,000 tonnes in Kenya, on an annual basis.

Gierend and Orr (2015) found that the consumer demand for millet rose with income group in all four countries. They attributed this to consumers’ broad appreciation of millet’s taste and nutritional value, with millet not considered an inferior good. The evidence was strongest in Tanzania, where millet consumption averaged 10 kg/capita in a high-income group compared to 3.2 kg/capita in a low-income group. In Kenya, the difference in consumption between high—and low-income groups was smallest, at 1.7 and 1.6 kg/capita, respectively.

## Consumption of millet in Uganda’s diet

This section starts with a brief description of Uganda’s broader food consumption patterns. It then presents the methods and data used to analyse an increase of millet consumption in the nation’s diet.

### Uganda food consumption patterns

National Panel Household Surveys (five waves conducted between 2009/10 and 2015/16), Demographic and Health Surveys, and data from the Agricultural Technology and Agribusiness Advisory Services, provide information on Ugandan food consumption. From these sources, the Ugandan National Planning Authority (2017) calculated that four out of ten Ugandans do not meet the minimum required energy intake of 2200 kcal per person per day, but instead consumed 1860 kcal per day on average.

Despite an overall reduction in child and adult undernutrition in recent years (World Food Programme 2013), 16% of Ugandan households as a whole remained food insecure in 2015/16 (compared to 20% in 2009/2010; Ugandan National Planning Authority 2017). Eastern Uganda however showed an increase in the prevalence of food insecurity between the earlier and later dates, rising from 33 to 46%. This was likely triggered by expansion in the production of crops such as sugar cane and rice as cash crops, along with decreasing farm sizes (Ugandan National Planning Authority 2017). Northern Uganda presented lower caloric intakes at the later of the two above dates, due primarily to seasonal food deficits aggravated by drought (Ugandan National Planning Authority 2017; Uganda Bureau of Statistics and World Food Programme 2013).

Despite Eastern and Northern Uganda regions’ worsening food security, they have higher dietary diversity than other parts of the country in terms of household consumption of food groups. At a national level, diet diversity for most Ugandans is below that recommended, though it did increase between 2009/10 and 2015/16 (Ugandan National Planning Authority 2017).

The most important staples in terms of caloric intake in Uganda are matooke, cassava and maize, followed by sweet potatoes and beans, with rice and wheat gaining prominence for urban high-income households. These staples are complemented by groundnut, sorghum, millet, Irish potatoes, peas, simsim (sesame) and green leafy vegetables; fruits, meat and milk are consumed on average twice a week (Ugandan National Planning Authority 2017). Overall, 69% of Ugandans’ food energy is derived from staples (Uganda Bureau of Statistics and World Food Programme 2013). The proportion varies between rural and urban areas, being 71% and 59%, respectively.

In Uganda, food insecurity and undernutrition are strongly correlated with monetary poverty; rural low-income households are therefore more likely to be food energy deficient, experience low dietary diversity, and depend on staples for energy (Uganda Bureau of Statistics and World Food Programme 2013).

This synopsis of the main facets of the Ugandan diet highlights the differentiation by rural/urban location and income as key factors, and this is reflected in our empirical analysis.

### Methods

To evaluate the effects of increasing the consumption of millet in the Ugandan diet we applied the model of Irz et al. (2015, 2016). They adapted the work by Jackson (1991) on generalized rationing theory, applying it to the case of linear constraints and extending it by deriving the comparative statistics necessary to empirically estimate healthy diets compatible with consumer preferences. For interested readers, Irz et al. (2015) made a comparison of the approach with other methods (e.g., nutrition linear programming models, demand systems) and explored the limitations of (e.g., nutrition linear programming models, demand systems) (pp. 189–90).

The starting point of the model is the neoclassical consumer theory that assumes that an individual chooses the consumption of $\mathrm{H}$ goods in quantities $\mathrm{x}=\left({\mathrm{x}}_{1},\ldots,{\mathrm{x}}_{\mathrm{H}}\right)$ to maximize a strictly increasing, strictly quasi-concave, twice differentiable utility function $\mathrm{U}=\mathrm{U}\left({\mathrm{x}}_{1},\ldots,{\mathrm{x}}_{\mathrm{H}}\right)$, subject to a linear budget constraint $\mathrm{p}\cdot \mathrm{x}\le \mathrm{M}$, where $\mathrm{p}$ is a price vector and $\mathrm{M}$ denotes income. In addition, departing from the standard model, the consumer faces $\mathrm{N}$ additional linear constraints.

These $\mathrm{N}$ constraints could for instance be maximum dietary intakes of nutrients such as salt, total fat, saturated fat or free sugars. Their linearity implies an assumption of constant nutritional coefficients for any food $\mathrm{i}$ and nutrient $\mathrm{n}$, the value of which is known from food composition tables. The constraints could also be food-based, such as recommendations on the consumption of fruit and vegetables. In this paper, an additional constraint related to the quantity of millet in the diet is considered.

The additional $N$ constraints are expressed as in Eq. (1):

1 $${\sum }_{\mathrm{i}=1}^{\mathrm{H}}{\mathrm{a}}_{\mathrm{i}}^{\mathrm{n}}{\mathrm{x}}_{\mathrm{i}}\le {\mathrm{r}}_{\mathrm{n}} ;\mathrm{ n}=1,\ldots,\mathrm{N}$$

The method to solve the modified utility maximisation problem relies on the notion of shadow prices, i.e., prices that would have to prevail for the unconstrained individual to choose the same bundle of goods as chosen when adding the constraints of Eq. (1). Duality theory is used to relate constrained and unconstrained problems in order to identify the properties of demand functions under additional constraints. Let the compensated (Hicksian) demand functions of the standard problem be ${\mathrm{h}}_{\mathrm{i}}\left(\mathrm{p},\mathrm{U}\right)$, and those of the constrained model ${\stackrel{\sim }{\mathrm{h}}}_{\mathrm{i}}\left(\mathrm{p},\mathrm{U},\mathrm{A},\mathrm{r}\right)$, where $\mathrm{A}$ is the $\left(\mathrm{N}\times \mathrm{H}\right)$ matrix of coefficients in (1), and $\mathrm{r}$ the N-vector of maximum amounts. By definition of the vector of shadow prices $\stackrel{\sim }{\mathrm{p}}$, the equality holds as in Eq. (2):

2 $${\stackrel{\sim }{\mathrm{h}}}_{\mathrm{i}}\left(\mathrm{p},\mathrm{U},\mathrm{A},\mathrm{r}\right)={\mathrm{h}}_{\mathrm{i}}\left(\stackrel{\sim }{\mathrm{p}},\mathrm{U}\right)$$

The minimum expenditure function of the constrained problem $\stackrel{\sim }{\mathrm{C}}\left(\mathrm{p},\mathrm{U},\mathrm{A},\mathrm{r}\right)$ can be related to the ordinary expenditure function $\mathrm{C}\left(\mathrm{p},\mathrm{U}\right)$ using Eq. (3):

3 $$\stackrel{\sim }{\mathrm{C}}\left(\mathrm{p},\mathrm{U},\mathrm{A},\mathrm{r}\right)={\sum }_{\mathrm{j}=1}^{\mathrm{H}}{\mathrm{p}}_{\mathrm{j}}{\stackrel{\sim }{\mathrm{h}}}_{\mathrm{j}}\left(\mathrm{p},\mathrm{U},\mathrm{A},\mathrm{r}\right)=\mathrm{C}\left(\stackrel{\sim }{\mathrm{p}},\mathrm{U}\right)+{\sum }_{\mathrm{j}=1}^{\mathrm{H}}\left({\mathrm{p}}_{\mathrm{j}}-{\stackrel{\sim }{\mathrm{p}}}_{\mathrm{j}}\right){\mathrm{h}}_{\mathrm{j}}\left(\stackrel{\sim }{\mathrm{p}},\mathrm{U}\right)$$

The constrained regime is fully characterised by the combination of the unconstrained demand functions, unconstrained expenditure function and the shadow prices. The shadow prices can be calculated based on the principle that they minimise the expenditure subject to the additional constraints. Thus, using Eq. (3), the Lagrange function of the constrained problem is given by Eq. (4):

4 $$\mathrm{L}=\mathrm{C}\left(\stackrel{\sim }{\mathrm{p}},\mathrm{U}\right)+{\sum }_{\mathrm{j}=1}^{\mathrm{H}}\left({\mathrm{p}}_{\mathrm{j}}-{\stackrel{\sim }{\mathrm{p}}}_{\mathrm{j}}\right){\mathrm{h}}_{\mathrm{j}}\left(\stackrel{\sim }{\mathrm{p}},\mathrm{U}\right)+{\sum }_{\mathrm{n}=1}^{\mathrm{N}}{\upmu }_{\mathrm{n}}\left({\mathrm{r}}_{\mathrm{n}}-{\sum }_{\mathrm{j}=1}^{\mathrm{H}}{\mathrm{a}}_{\mathrm{j}}^{\mathrm{n}}{\mathrm{h}}_{\mathrm{j}}\left(\stackrel{\sim }{\mathrm{p}},\mathrm{U}\right)\right)$$

Assuming non-satiation so that all the shadow prices are positive, the Kuhn-Tucker conditions derived from Eq. (4) are in Eqs. (5) and (6):

5 $$\frac{{\partial {\text{L}}}}{{\partial {\tilde{\text{p}}}_{{\text{i}}} }} = \frac{{\partial {\text{C}}}}{{\partial {\tilde{\text{p}}}_{{\text{i}}} }} - {\text{h}}_{{\text{i}}} + \mathop \sum \limits_{{{\text{j}} = 1}}^{{\text{H}}} \left( {{\text{p}}_{{\text{j}}} - {\tilde{\text{p}}}_{{\text{j}}} } \right)\frac{{\partial {\text{h}}_{{\text{j}}} }}{{\partial {\tilde{\text{p}}}_{{\text{i}}} }} - \mathop \sum \limits_{{{\text{n}} = 1}}^{{\text{N}}} {\upmu }_{{\text{n}}} \left( {\mathop \sum \limits_{{{\text{j}} = 1}}^{{\text{H}}} {\text{a}}_{{\text{j}}}^{{\text{n}}} \frac{{\partial {\text{h}}_{{\text{j}}} }}{{\partial {\tilde{\text{p}}}_{{\text{i}}} }}} \right) = 0,{\text{ i}} = 1, \ldots ,{\text{H}}\frac{{\partial {\text{L}}}}{{\partial {\tilde{\text{p}}}_{{\text{i}}} }} = \frac{{\partial {\text{C}}}}{{\partial {\tilde{\text{p}}}_{{\text{i}}} }} - {\text{h}}_{{\text{i}}} + \mathop \sum \limits_{{{\text{j}} = 1}}^{{\text{H}}} \left( {{\text{p}}_{{\text{j}}} - {\tilde{\text{p}}}_{{\text{j}}} } \right)\frac{{\partial {\text{h}}_{{\text{j}}} }}{{\partial {\tilde{\text{p}}}_{{\text{i}}} }} - \mathop \sum \limits_{{{\text{n}} = 1}}^{{\text{N}}} {\upmu }_{{\text{n}}} \left( {\mathop \sum \limits_{{{\text{j}} = 1}}^{{\text{H}}} {\text{a}}_{{\text{j}}}^{{\text{n}}} \frac{{\partial {\text{h}}_{{\text{j}}} }}{{\partial {\tilde{\text{p}}}_{{\text{i}}} }}} \right) = 0,{\text{ i}} = 1, \ldots ,{\text{H}}\frac{{\partial {\text{L}}}}{{\partial {\tilde{\text{p}}}_{{\text{i}}} }} = \frac{{\partial {\text{C}}}}{{\partial {\tilde{\text{p}}}_{{\text{i}}} }} - {\text{h}}_{{\text{i}}} + \mathop \sum \limits_{{{\text{j}} = 1}}^{{\text{H}}} \left( {{\text{p}}_{{\text{j}}} - {\tilde{\text{p}}}_{{\text{j}}} } \right)\frac{{\partial {\text{h}}_{{\text{j}}} }}{{\partial {\tilde{\text{p}}}_{{\text{i}}} }} - \mathop \sum \limits_{{{\text{n}} = 1}}^{{\text{N}}} {\upmu }_{{\text{n}}} \left( {\mathop \sum \limits_{{{\text{j}} = 1}}^{{\text{H}}} {\text{a}}_{{\text{j}}}^{{\text{n}}} \frac{{\partial {\text{h}}_{{\text{j}}} }}{{\partial {\tilde{\text{p}}}_{{\text{i}}} }}} \right) = 0,{\text{ i}} = 1, \ldots ,{\text{H}}$$

6 $$\begin{aligned} \frac{{\partial {\text{L}}}}{{\partial {\upmu }_{{\text{n}}} }} & = {\upmu }_{{\text{n}}} \left( {{\text{r}}_{{\text{n}}} - \mathop \sum \limits_{{{\text{j}} = 1}}^{{\text{H}}} {\text{a}}_{{\text{j}}}^{{\text{n}}} {\text{h}}_{{\text{j}}} } \right) = 0,{\text{ n}} = 1,\ldots,{\text{N}} \\ & \quad \quad \quad \quad {\upmu }_{{\text{n}}} \ge 0,{\text{n}} = 1,\ldots,{\text{N}} \\ \end{aligned}\begin{aligned} \frac{{\partial {\text{L}}}}{{\partial {\upmu }_{{\text{n}}} }} & = {\upmu }_{{\text{n}}} \left( {{\text{r}}_{{\text{n}}} - \mathop \sum \limits_{{{\text{j}} = 1}}^{{\text{H}}} {\text{a}}_{{\text{j}}}^{{\text{n}}} {\text{h}}_{{\text{j}}} } \right) = 0,{\text{ n}} = 1,\ldots,{\text{N}} \\ & \quad \quad \quad \quad {\upmu }_{{\text{n}}} \ge 0,{\text{n}} = 1,\ldots,{\text{N}} \\ \end{aligned}\begin{aligned} \frac{{\partial {\text{L}}}}{{\partial {\upmu }_{{\text{n}}} }} & = {\upmu }_{{\text{n}}} \left( {{\text{r}}_{{\text{n}}} - \mathop \sum \limits_{{{\text{j}} = 1}}^{{\text{H}}} {\text{a}}_{{\text{j}}}^{{\text{n}}} {\text{h}}_{{\text{j}}} } \right) = 0,{\text{ n}} = 1,\ldots,{\text{N}} \\ & \quad \quad \quad \quad {\upmu }_{{\text{n}}} \ge 0,{\text{n}} = 1,\ldots,{\text{N}} \\ \end{aligned}$$

Using Shephard’s lemma and denoting $\partial {\mathrm{h}}_{\mathrm{i}}/\partial {\mathrm{p}}_{\mathrm{j}}$ (i.e., the Slutsky term) by ${\mathrm{s}}_{\mathrm{ij}}$, Eq. (5) becomes (7):

7 $${\sum }_{\mathrm{j}=1}^{\mathrm{H}}\left[\left({\mathrm{p}}_{\mathrm{j}}-{\stackrel{\sim }{\mathrm{p}}}_{\mathrm{j}}\right)-{\sum }_{\mathrm{n}=1}^{\mathrm{N}}{\upmu }_{\mathrm{n}}{\mathrm{a}}_{\mathrm{j}}^{\mathrm{n}}\right]{\mathrm{s}}_{\mathrm{ji}}=0,\mathrm{ i}=1,\ldots,\mathrm{H}$$

For Eq. (7) to hold it is necessary for the term in brackets to be equal to zero. Assuming all the $\mathrm{N}$ constraints are binding, the shadow price problem in Eq. (6) reduces to Eqs. (8):

8 $$\begin{array}{c}{\stackrel{\sim }{\mathrm{p}}}_{\mathrm{i}}={\mathrm{p}}_{\mathrm{i}}-{\sum }_{\mathrm{n}=1}^{\mathrm{N}}{\upmu }_{\mathrm{n}}{\mathrm{a}}_{\mathrm{i}}^{\mathrm{n}},\mathrm{i}=1,\ldots,\mathrm{H}\\ {\sum }_{\mathrm{i}=1}^{\mathrm{H}}{\mathrm{a}}_{\mathrm{i}}^{\mathrm{n}}{\mathrm{h}}_{\mathrm{i}}\left(\stackrel{\sim }{\mathrm{p}},\mathrm{U}\right)={\mathrm{r}}_{\mathrm{n}},\mathrm{n}=1,\ldots,\mathrm{N}\end{array}$$

Due to its nonlinear nature, Eq. (8) cannot be solved analytically; however, Irz et al. (2015) provide a method where the solution can be computed iteratively. In fact, they simulate the impact of adopting recommendations in a Marshallian context, i.e., holding income (or total expenditure) and prices constant. The structure of the solution procedure is as follows:

- Given a percentage change in the level of the additional constraints (i.e., ${\mathrm{r}}_{\mathrm{n}}$), the changes in Hicksian demands are calculated ($\partial \mathrm{h}/\partial \mathrm{r}$).
- The quantities (i.e., Hicksian quantities) thus are obtained and the original prices are then combined to calculate the compensating variation (CV) associated with the imposition of the additional constraints. The CV is given by $\mathrm{C}\left(\mathrm{p},\mathrm{U}\right)-\stackrel{\sim }{\mathrm{C}}\left(\mathrm{p},\mathrm{U},\mathrm{A},\mathrm{r}\right)=-{\sum }_{\mathrm{n}=1}^{\mathrm{N}}{\sum }_{\mathrm{i}=1}^{\mathrm{H}}{\mathrm{p}}_{\mathrm{i}}\left(\frac{\partial {\mathrm{h}}_{\mathrm{i}}}{\partial {\mathrm{r}}_{\mathrm{n}}}\right)$.
- The CV, which hypothetically would allow the consumer to maintain their utility level, is then removed to calculate the corresponding changes in the Marshallian demand (i.e., $\mathrm{\Delta x}$), as in equation $\mathrm{\Delta x}=\mathrm{\Delta h}=\stackrel{\sim }{\mathrm{h}}\cdot\upeta \cdot \frac{\mathrm{CV}}{\mathrm{p}\cdot \stackrel{\sim }{\mathrm{h}}}$.
- Note that because the additional constraint is directly imposed on the Hicksian demands rather than the Marshallian ones, there is no guarantee that the diets calculated in step c will satisfy the constraints. Therefore, there is the need to evaluate the constraints using the resulting Marshallian demands.
- If this Marshallian solution satisfies the constraints, then the procedure is completed. If the solution does not satisfy the recommendation, the changes in the constraints need to be adjusted in the first step and the procedure run again.

In addition to solving the consumer problem of including higher quantities of millet in the diet (through increasing the recommended quantity of millet), this study also estimated the change in the nutritional value of the diet (in contrast to the nutritional value of millet alone). This was done in two steps: first, once the new consumption was computed, it was transformed into its nutritional components using nutritional coefficients. Second, in order to summarise the nutritional results, the Mean Adequacy Ratio (MAR) was computed. This estimates the percentage of mean daily intake of beneficial nutrients, with 100% representing a diet which would conform to all nutritional requirements (Vieux et al. 2013). The nutrients used here were chosen based on data availability; they included calcium, iron, zinc, vitamin C, thiamine (vitamin B1), riboflavin (vitamin B2), niacin (vitamin B3), vitamin B6, folate and vitamin A. Note that the components of the MAR are truncated to 100, so surpluses of any nutrient cannot compensate the lack of another. The formula of the MAR is given by (9), where ${\mathrm{c}}_{\mathrm{i}}$ is the intake of nutrient i and ${\mathrm{R}}_{\mathrm{i}}$ its recommended intake.^2^

9 $$\mathrm{MAR}=\frac{1}{10}\times \sum_{\mathrm{i}=1}^{10}\frac{{\mathrm{c}}_{\mathrm{i}}}{{\mathrm{R}}_{\mathrm{i}}}\times 100$$

To summarise, the method extends the theory of the consumer under rationing and shows that adjustments in consumption can be estimated by combining data on food consumption, price (Hicksian and Marshallian) and expenditure elasticities, as well as food composition data.

The next section presents the application of this method to the case of increasing the consumption of millet in the Ugandan diet.

### Data and implementation

Given the varying consumption characteristics of Uganda’s population, the use of average elasticities, quantities, prices and food composition information is not the best approach; we therefore considered three differentiated cases: rural consumers, poor urban consumers (urban-poor) and affluent urban consumers (urban-affluent).

Most of the required information (demand elasticities and expenditure) for the three aforementioned groups was obtained from Boysen (2016), who estimated unconditional (Marshallian and Hicksian) own price elasticities and income (expenditure) elasticities for Uganda by expenditure quintile using a two-stage budgeting demand system including one non-food and 14 food items based on the 2012/2013 Ugandan National Household Survey (UNHS), a nationally representative survey of 6887 households.^3^ In the first stage households allocate their consumption budget to food and non-food items. In the second budgeting stage, households allocate the food budget to 14 different item groups. Then, the three aforementioned consumer groups were established using the average for the rural consumers’ group, average of information for the lower three urban quintiles for the poor urban consumers group, and the average of the top two quintiles for the affluent urban consumers group).

**Fig. 2:**
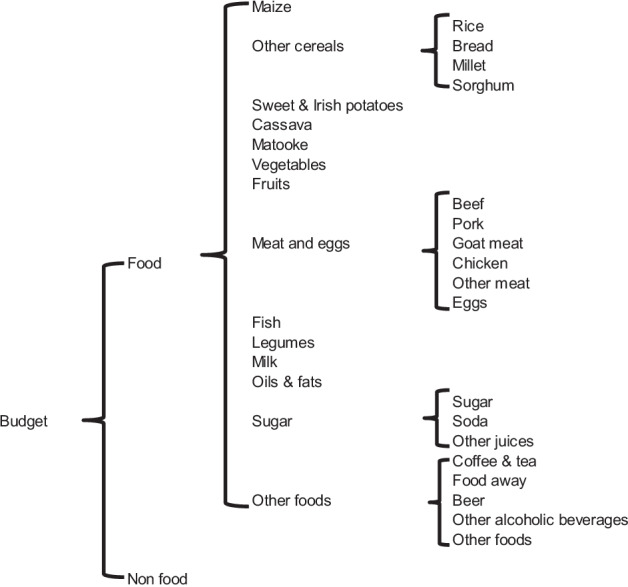
Uganda augmented demand system. *Source*: Based on Boysen (2016)

The classification used in Boysen (2016) does not fully fit the purpose of this study due to the fact that millet is aggregated with other cereals in the groups of “other cereals”. To address this, an additional budget stage was added by estimating several conditional demand systems and using the method in Carpentier and Guyomard (2001) to compute the unconditional budgetary third stage.^4^ The structure of the final demand system considers a total of 28 categories is presented in Fig. 2.

The nutritional analysis (e.g., for the computation of the MAR) requires actual quantities and prices. A limitation of the Living Standard Measurement Surveys for Uganda and many other countries is that the recorded quantities are not uniform (e.g., quantities are recorded in the measurement scale provided by the interviewee and this can be small, medium or large buckets, heaps or clusters among others), whilst the nutritional information is provided for specific weights (e.g., per 100 g).^5^ The approach adopted was to use the retail prices recorded by product provided by the Uganda Bureau of Statistics (UBOS) (2013), which cover the period analysed by Boysen (2016) and six price collection points in the country. These prices, expressed in Uganda Shillings (UGX) per metric unit, were used to obtain the quantities consumed within each one of the three groups analysed (Table 1).

**Table 1:** Uganda—consumption by socioeconomic group. *Sources*: Boysen (2016), UBOS (2013)

Goods	Units	Rural						Urban-lower quintiles						Urban-upper quintiles					
		Quantities	Prices	Total expend. UGX	Own-Price Elasticity	Income Elastic	Food shares	Quantities	Prices	Total Expend. UGX	Own-Price Elasticity	Income Elastic	Food shares	Quantities	Prices	Total Expend. UGX	Own-Price Elasticity	Income Elastic	Food shares
Millet	(Kg)	6.6	1506.0	9875.1	− 1.228	0.950	0.0160	5.0	1506.0	7483.1	− 1.011	0.596	0.0137	8.0	1506.0	12,016.2	− 1.014	0.405	0.0090
Maize	(Kg)	39.3	1616.8	63,571.2	− 1.450	1.010	0.1030	31.4	1717.1	53,841.6	− 1.427	0.713	0.0983	31.4	1890.8	59,413.2	− 1.730	0.430	0.0445
Rice	(Kg)	3.1	3361.9	10,492.3	− 1.207	0.949	0.0170	6.1	3369.5	20,624.1	− 0.976	0.596	0.0377	11.2	5497.4	61,415.9	− 0.977	0.405	0.0460
Bread	(500 g)	2.5	1962.2	4937.6	− 1.233	0.951	0.0080	5.9	1975.0	11,680.9	− 1.193	0.598	0.0213	32.7	1855.3	60,748.4	− 1.344	0.406	0.0455
Sorghum	(Kg)	9.9	1615.0	16,047.1	− 1.319	0.950	0.0260	4.7	1703.2	8030.6	− 0.992	0.596	0.0147	1.4	1397.5	2002.7	− 1.013	0.404	0.0015
Sweet & Irish potatoes	(Kg)	109.6	625.0	68,508.8	− 0.990	1.030	0.1110	80.4	658.5	52,929.0	− 0.703	0.773	0.0967	79.0	752.1	59,413.2	− 0.405	0.500	0.0445
Cassava	(Kg)	94.0	807.4	75,915.2	− 0.570	0.550	0.1230	47.8	816.5	39,057.9	− 0.690	0.613	0.0713	38.3	836.8	32,043.1	− 0.210	0.485	0.0240
Matooke	(Kg)	95.5	575.3	54,930.5	− 0.950	1.530	0.0890	89.5	522.1	46,723.5	− 1.263	1.263	0.0853	229.4	555.8	127,504.8	− 0.905	0.715	0.0955
Vegetables	(Kg)	42.1	1011.3	42,586.6	− 0.470	0.050	0.0690	32.2	1216.6	39,130.9	− 0.637	0.427	0.0715	47.0	1313.0	61,683.0	− 0.470	0.225	0.0462
Fruits	(Kg)	18.9	1110.4	20,984.7	− 1.210	1.240	0.0340	16.1	1202.2	19,346.5	− 0.633	1.073	0.0353	37.5	1636.0	61,415.9	− 0.620	0.845	0.0460
Beef	(Kg)	3.1	7268.2	22,219.1	− 1.082	1.919	0.0360	3.4	7331.3	24,986.1	− 0.881	1.869	0.0456	13.0	7541.9	98,065.2	− 0.453	1.074	0.0735
Pork	(Kg)	0.7	8304.2	6172.0	− 1.114	1.920	0.0100	0.7	8021.0	5292.9	− 0.916	1.870	0.0097	1.6	7467.4	12,283.2	− 0.908	1.075	0.0092
Goat meat	(Kg)	0.9	8633.3	7406.4	− 1.106	1.921	0.0120	0.6	8638.2	5292.9	− 0.912	1.871	0.0097	2.0	8836.5	18,024.2	− 0.893	1.075	0.0135
Chicken	(Kg)	1.5	9955.1	14,812.7	− 1.122	1.920	0.0240	0.9	10,280.4	9490.7	− 0.873	1.870	0.0173	3.6	10,237.0	37,183.3	− 0.824	1.075	0.0279
Other meat	(Kg)	0.1	10,000.0	1234.4	− 1.119	1.917	0.0020	0.1	10,000.0	730.1	− 0.890	1.865	0.0013	0.3	10,000.0	2670.3	− 0.344	1.072	0.0020
Eggs	(2 eggs)	4.1	599.6	2468.8	− 1.139	1.934	0.0040	4.9	596.3	2920.2	− 0.994	1.880	0.0053	27.4	597.3	16,355.3	− 0.949	1.079	0.0123
Fish	(Kg)	2.0	12,467.7	25,305.1	− 1.370	1.620	0.0410	1.7	14,201.9	24,456.8	− 1.330	1.513	0.0447	4.7	12,076.1	56,743.0	− 1.250	1.045	0.0425
Pulses, legumes, nuts	(Kg)	27.5	2675.4	73,569.8	− 0.760	0.660	0.1192	25.3	2629.4	66,416.7	− 0.837	0.617	0.1213	32.5	2913.3	94,794.2	− 0.765	0.360	0.0710
Milk	(Ltr)	17.8	1054.4	18,762.8	− 1.420	1.550	0.0304	19.4	1030.7	19,967.0	− 1.400	1.383	0.0365	61.5	1041.9	64,086.2	− 1.180	0.755	0.0480
Oils & fats	(300 ml)	4.2	3096.2	12,961.1	− 0.610	0.770	0.0210	7.9	2075.5	16,316.7	− 0.673	0.717	0.0298	12.8	2712.7	34,713.4	− 0.560	0.375	0.0260
Sugar	(Kg)	3.8	5368.9	20,367.5	− 0.946	1.170	0.0330	4.9	5477.4	26,993.8	− 1.038	0.747	0.0493	11.6	5447.8	63,418.6	− 0.975	0.545	0.0475
Soda	(300 ml)	3.1	1000.0	3086.0	− 0.965	1.170	0.0050	3.5	1000.0	3467.8	− 0.973	0.746	0.0063	29.4	1000.0	29,372.8	− 0.977	0.545	0.0220
Other juices	(Ltr)	0.0	1031.7	0.6	− 0.818	1.174	0.0000	1.0	951.4	912.6	− 0.982	0.747	0.0017	5.6	961.7	5340.5	− 0.988	0.545	0.0040
Coffee & tea	(Kg)	15.5	79.7	1234.4	− 0.947	1.077	0.0020	24.9	73.9	1843.4	− 0.927	1.360	0.0034	29.7	159.8	4739.7	− 0.845	1.099	0.0036
Food away	(Kg)	16.4	1,031.7	16,911.2	− 1.565	1.081	0.0274	25.9	951.4	24,621.1	− 0.887	1.364	0.0450	223.5	961.7	214,955.8	− 0.899	1.106	0.1610
Beer	(500 ml)	1.0	2436.1	2468.8	− 0.683	1.078	0.0040	0.7	2423.0	1642.6	− 0.733	1.359	0.0030	8.4	2381.9	20,026.9	− 0.291	1.103	0.0150
Other alcoholic beverages	(300 ml)	9.2	1003.1	9257.9	− 1.802	1.080	0.0150	2.9	1002.9	2920.2	− 2.654	1.365	0.0053	4.7	1003.1	4673.0	− 7.493	1.110	0.0035
Other foods	(500 g)	8.7	1270.4	11,109.5	− 1.348	1.078	0.0180	34.8	299.3	10,421.5	− 0.946	1.360	0.0190	7.5	2679.7	20,026.9	− 1.005	1.097	0.0150
Annual per capita expenditure (000 UGX)		1094.3						1107.1						4055.7					

The quantities are per capita per year

The method by Irz et al. (2015) requires the full matrices of price elasticities, while Boysen (2016) provides only own-price and expenditure elasticities. Cross-price elasticities were calibrated using Beghin et al. (2004) approach, which allows their computation within a demand system that is theoretically consistent with consumer theory.

Beghin et al. (2004) method is a flexible calibration technique for partial demand systems, combining the developments in incomplete demand systems (LaFrance and Hanemann 1989; LaFrance 1998) with a set of restrictions conditioned on the available elasticity estimates. The technique accommodates various degrees of knowledge on cross-price elasticities, satisfies curvature restrictions, and allows the recovery of an exact welfare measure for policy analysis (i.e., the equivalent variation).

An overview of the calibration procedure is provided below, more detail is available in Beghin et al. (2004). The calibration approach builds on the Linquad structure (LaFrance et al. 2002) as the foundation for the partial demand system. The Linquad demand system is generated from the following expenditure function,$\mathrm{C}\left(\mathrm{p},{\mathrm{p}}_{\mathrm{z}},\uptheta \right)$^6^ (10):

10 $$\mathrm{C}\left(\mathrm{p},{\mathrm{p}}_{\mathrm{z}},\uptheta \right)=\mathrm{p{^{\prime}}}\upvarepsilon +\frac{1}{2}\mathrm{p{^{\prime}}}\mathrm{Vp}+\updelta \left({\mathrm{p}}_{\mathrm{z}}\right)+\uptheta \left({\mathrm{p}}_{\mathrm{z}},\mathrm{U}\right){\mathrm{e}}^{{\upchi }^{\mathrm{i}}\mathrm{p}}$$

where $\updelta \left({\mathrm{p}}_{\mathrm{z}}\right)$ is an arbitrary real value function of ${\mathrm{p}}_{\mathrm{z}}$, i.e. the prices of all the other goods not considered on the incomplete demand system; $\uptheta \left({\mathrm{p}}_{\mathrm{z}},\mathrm{U}\right)$ is the constant of integration, which is increasing in U; and $\upchi ,\upvarepsilon$ and $VVV$ are the vectors and respectively the matrix of parameters to be recovered in the calibration. Applying Shepherd’s lemma to Eq. (10), the Hicksian demand function is obtained (11):

11 $$\mathrm{h}=\upvarepsilon +\mathrm{Vp}+\upchi \left[\uptheta \left({\mathrm{p}}_{\mathrm{z}},\mathrm{U}\right){\mathrm{e}}^{{\upchi }^{\mathrm{i}}\mathrm{p}}\right]$$

The integrating factor, $\uptheta \left({\mathrm{p}}_{\mathrm{z}},\mathrm{U}\right){\mathrm{e}}^{{\upchi }^{\mathrm{i}}\mathrm{p}}$, makes the demand system an exact system of partial differential equations. The Linquad expenditure function (10) provides a complete solution class to this system of differentials and represents the exhaustive class of expenditure functions generating demands for quantities x that are linear in total income (M) and linear and quadratic in prices for x.

Solving the expenditure function (10) for $\uptheta \left({\mathrm{p}}_{\mathrm{z}},\mathrm{U}\right){\mathrm{e}}^{{\upchi }^{\mathrm{i}}\mathrm{p}}$ and replacing expenditure with M for income yields the Linquad Marshallian demand functions (12):

12 $$\mathrm{x}=\upvarepsilon +\mathrm{Vp}+\upchi \left(\mathrm{M}-\mathrm{\varepsilon {^{\prime}}}\mathrm{p}-\frac{1}{2}\mathrm{p{^{\prime}}}\mathrm{Vp}-\updelta \left({\mathrm{p}}_{\mathrm{z}}\right)\right)$$

Then, the Marshallian price elasticities ($\varepsilon_{ij}\varepsilon_{ij}\varepsilon_{ij}$) are given by (13):

13 $${\upvarepsilon }_{\mathrm{ij}}=\left[{\upnu }_{\mathrm{ij}}-{\upchi }_{\mathrm{i}}\left({\upvarepsilon }_{\mathrm{j}}+{\sum }_{\mathrm{k}=1}^{\mathrm{H}}{\upnu }_{\mathrm{jk}}{\mathrm{p}}_{\mathrm{k}}\right)\right]{\mathrm{p}}_{\mathrm{j}}/{\mathrm{x}}_{\mathrm{i}}\mathrm{ i}=1,\ldots,\mathrm{H};\mathrm{j}=1,\ldots,\mathrm{H}$$

The Slutsky matrix (S) is given by (14):

14 $$\mathrm{S}=\mathrm{V}+\left(\mathrm{M}-\mathrm{\varepsilon {^{\prime}}}\mathrm{p}-\frac{1}{2}\mathrm{p{^{\prime}}}\mathrm{Vp}-\updelta \left({\mathrm{p}}_{\mathrm{z}}\right)\right)\mathrm{\chi \chi {^{\prime}}}$$

The Hicksian elasticities (${\upvarepsilon }_{\mathrm{ij}}^{\mathrm{h}}$) are given by (15):

15 $${\upvarepsilon }_{\mathrm{ij}}^{\mathrm{h}}=\left[{\upnu }_{\mathrm{ij}}-{\upchi }_{\mathrm{i}}{\upchi }_{\mathrm{j}}\left(\mathrm{M}-\mathrm{\varepsilon {^{\prime}}}\mathrm{p}-\frac{1}{2}\mathrm{p{^{\prime}}}\mathrm{Vp}-\updelta \left({\mathrm{p}}_{\mathrm{z}}\right)\right)\right]{\mathrm{p}}_{\mathrm{j}}/{\mathrm{x}}_{\mathrm{i}}\mathrm{i}=1,\ldots,\mathrm{H};\mathrm{j}=1,\ldots,\mathrm{H}$$

The necessary information set for the calibration is as follows: income and own-price elasticity estimates, levels of Marshallian demands x, the total income (or expenditure M) and prices. This assumes that no cross-price elasticities are available and thus need to be computed.

The calibration involves the recovery of elements of the H-vectors, $\upchi$ and $\upvarepsilon$, together with the elements of the $\mathrm{H}\times \mathrm{H}$ matrix (i.e., ${\upnu }_{\mathrm{ij}}$). The calibration imposes symmetry and negative semi-definiteness of the Hessian of the expenditure function. In the demand system, homogeneity degree 1 in prices for the expenditure function is imposed by deflating prices by a consumer price index although homogeneity in prices plays no role in the recovery of parameters in the calibration procedure.

The calibration is done sequentially. First, point estimates of derivatives of demand with respect to income $\upchi$ are obtained from the known income elasticity estimates such as ${\upchi }_{\mathrm{i}}={\mathrm{x}}_{\mathrm{i}}{\upeta }_{\mathrm{i}/\mathrm{M}}$. Then, income response parameters are substituted into (12) and (13). Next, price responses are recovered from the point estimates corresponding to the available price elasticities, evaluated at the reference level of the data. Then, all price responses together with restrictions on S from integrability, and the observed demanded quantities are used to estimate the remaining parameters of the demand system.

Both procedures, the augmented microeconomic consumer problem and the calibration of elasticities were implemented in MS Excel by means of routines written in Visual Basic for Application (VBA). The calibrated Marshallian and Hicksian price elasticity matrices by consumer group (Additional file 1: Tables A1 to A3), and the food composition information (Additional file 1: Table A4) are presented in the Annex.

## Results and discussion

The implemented simulation model was used for two simulations that aimed to increase the amount of millet in the diets of the three consumer groups by 50 per cent and, respectively, by 100% (i.e., double it). Note that on the one hand, although these percentages might appear large, the quantities of millet in the diet are small, so the actual increase is realistic. On the other hand, large increases in the demand for millet are needed to motivate expansion of the supply of millet.

**Fig. 3:**
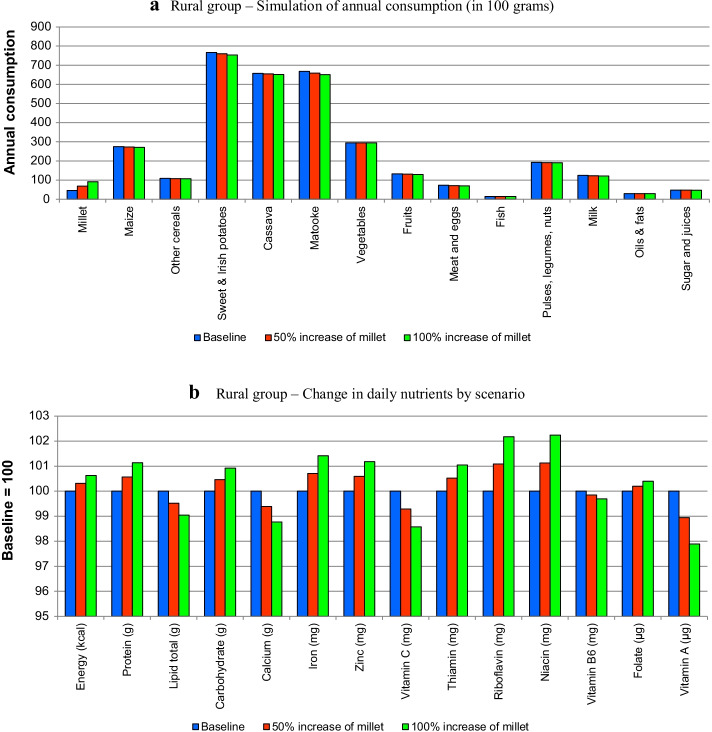
**a** Rural group—simulation of annual consumption (in 100 g). **b** Rural group—change in daily nutrients by scenario

Figure 3a, b present the simulation results for the rural group, 4a and 4b for the poor urban group and 5a and 5b for the affluent urban groups. They present the changes in quantities in the diets and changes in nutrients.

**Fig. 4:**
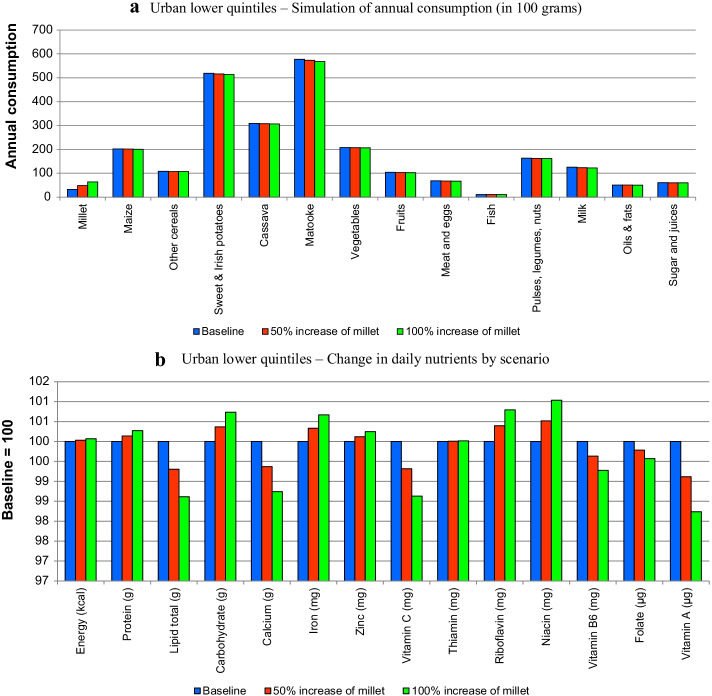
**a** Urban lower quintiles—simulation of annual consumption (in 100 g). **b** Urban lower quintiles—change in daily nutrients by scenario

**Fig. 5:**
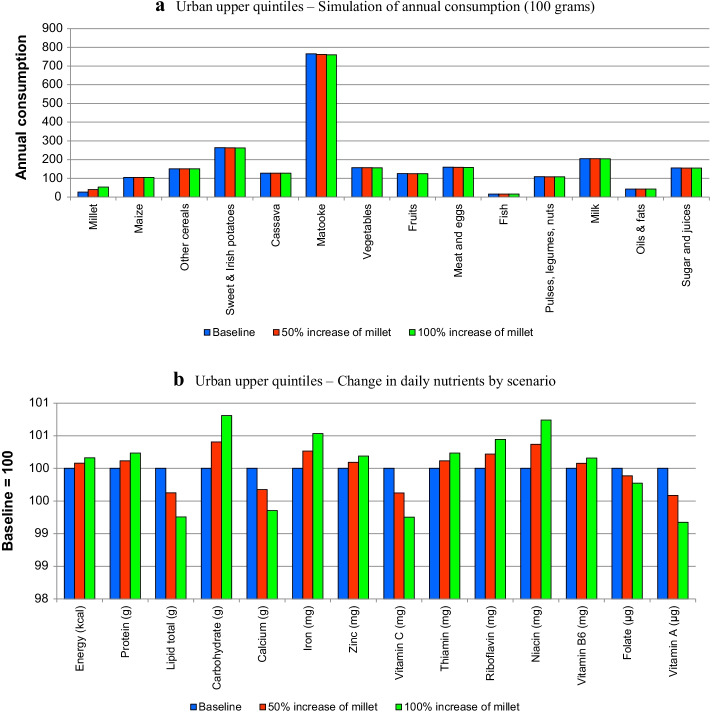
**a** Urban upper quintiles—simulation of annual consumption (100 g). **b** Urban upper quintiles—change in daily nutrients by scenario

The changes in quantities (i.e., Figs. 3a, 4a, 5a) show the rise of millet, which, despite the high simulated increases, still represents a small percentage in the diet in comparison with other staples such as matooke or maize. Note that the higher quantity of millet in the diet brings dietary changes due to two reasons, namely preferences for different foods and the fact that they compete within the consumer budget.

Figures 3a, 4a and 5a show that most of the changes occur in the quantities of staples (maize, other cereals, potatoes and matooke), although the other foods are also slightly affected. This is common for all the groups, although, as shown in Fig. 5a, the changes are less significant.

Figures 3b, 4b, 5b show the changes in nutrients in terms of indices (with respect to baseline equal to 100). All figures show that the introduction of millet increases the amount of calories in the diet with respect to the baseline. In terms of macronutrients (i.e., proteins, lipids and carbohydrates) the results indicate that with the exception of lipids, millet contributes positively to the diet of all population groups. These are deficiencies that have been mentioned in the literature, particularly regarding women (e.g., Bachou and Labadarios 2002).

With respect to micronutrients, the results indicate that the expansion of millet in the diet improves the quantities of iron, zinc, riboflavin and niacin in all groups. It, however, has negative effects on calcium, vitamin C, and vitamin A in all groups. The remaining micronutrients (i.e., thiamine, vitamin B6 and folate) showed differences by group. Thus, thiamine increased in the rural and affluent urban group, whilst decreasing on the poor urban group; vitamin B6 decreased in the rural and poor urban group and increased in the affluent urban group, and folate decreased in the urban groups and increased in the rural group.

USAID (2014) points out micronutrient deficiencies in Uganda, particularly vitamin A and iron, which are highly prevalent in women and children. The results indicate that, whilst iron may increase with more millet, vitamin A decreases for all groups. The latter finding might be explained by the reduction of matooke in the diet, which, as shown by the food composition table (Additional file 1: Table A4 in the Annex), brings vitamin A to the diet, whilst millet does not.

**Fig. 6:**
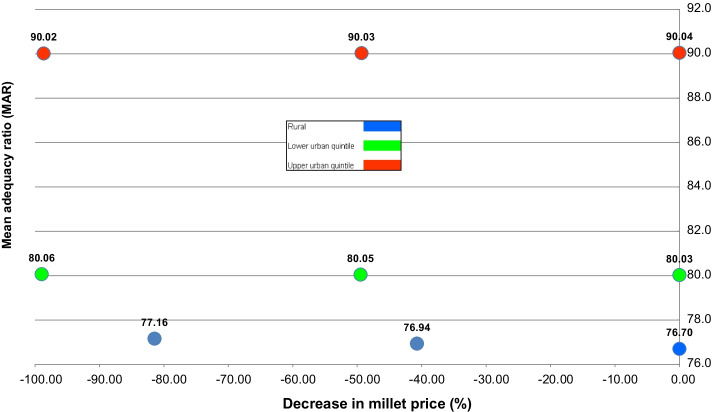
Decrease in price and MAR due to increase of millet in the diet

Figure 6 summarises simulations for the three consumer groups by presenting the relationship between the change in the price of millet required to increase the quantity demanded, as simulated, and the MAR as a measure of nutritional quality. As the increase in the quantity of millet relates to a decrease in price, the current baseline is located at the right-hand side of the figure; results for the 50% increase in the quantity of millet are in the middle; and results for the 100% increase in the quantity of millet are at the left-hand side. As millet consumption expands (move from right to left of the figure), the rural poor benefit more than other consumers according to the MAR indicator, though for all consumer groups the changes are only marginal; for the urban-affluent group, the MAR slightly decreases with the increases in millet consumption.

In regard to the changes in the millet prices required by the simulations, the rural group needed the least reduction to increase their consumption. Nevertheless, the simulations indicated that to increase the quantity of millet in the diet under current consumers’ preferences would require substantive reductions in price. Below, we discuss the implications of these findings further.

The first point is that our simulations are a reminder that the evaluation of the nutritional benefits of the expansion of the consumption of any food product needs to be placed within the context of the broader diet because of displacement effects. In our study this effect explained the low MAR indicators and some of the conflicting nutritional results presented in Figs. 3, 4 and 5. This indicates that blanket recommendations to increase millet in the diet based only on its nutritional characteristics and in comparison only with major staples is inappropriate.

The second point relates to the significant price reductions (comparing the shadow price with the original price) that would be needed to markedly increase millet consumption. This would require expanding the productivity of supply considerably, which is unrealistic. Likely more important therefore is the need to expand actual demand by measures such as improving consumers’ appreciation for millet (i.e., their willingness to pay). This could involve enhancing the quality of millet products and the introduction of well-targeted new products incorporating millet, that reflect consumers’ tastes and are made accessible to them. One way could be to promote the crop as an ingredient replacing or complementing other cereals in the preparation of foods such as breakfast cereals or biscuits.^7^

It is important to stress however that only after considering consumers’ views of millet will it be possible to envisage a strategy to restore it to its previous importance in the Ugandan diet. This is akin to collecting ethnographic evidence related to millet use in India (Chera 2017). It is already known that subnational cultural and social factors are important determinants of millet consumption in Uganda (USAID 2014), so different approaches to stimulate consumption may be required in different parts of the country.

**Fig. 7:**
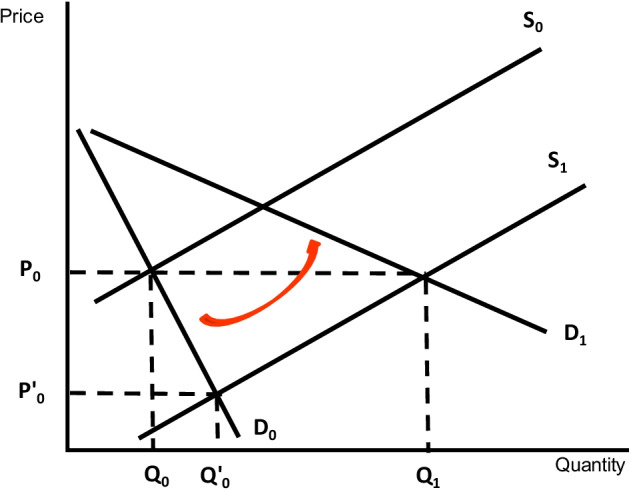
Need of a multidisciplinary approach to expand the consumption of millet

In Fig. 7 we further illustrate the relationship between price and the quantity of consumption. In the diagram, the original equilibrium point is given by *P*_0_, *Q*_0_. Given current preferences (i.e., the demand schedule measuring the total demand of the crop), a supply policy increasing the productivity of the crop that reduces the marginal costs of production would move supply from *S*_0_ to *S*_1_. This would require a substantive reduction in the marginal costs to obtain an increase in the equilibrium quantity from *Q*_0_ to *Q*’_0_ (almost twice the demanded quantity) and a substantive reduction in market price from *P*_0_ to *P*’_0_. Instead, a more effective way to achieve the target of expanding the consumption of the crop would be to consider way to expand the demand. This is by increasing the appreciation of consumers for the crop as measured by willingness to pay (the rotation outward of the demand curve) could keep the original price but expand demand to Q_1_.

## Conclusions

Underutilized, neglected or orphan crops have been cited as having the potential to play a number of roles in the improvement of food and nutritional security; however, consumers in developing countries are also increasingly abandoning their traditional diets and replacing them with ‘western’ ones based on a small set of major staples.

In this paper, we have investigated the implications of expanding the consumption of underutilized crops on current diets. We did so by considering consumers’ preferences in the form of price and income elasticities, using a modified version of the microeconomic consumer problem, augmented with linear constraints based on the generalized rationing theory. The method was applied to the case study of the consumption of millet in Uganda by three socioeconomic groups.

Our results show complex impacts on dietary quality. They also show that the introduction of millet increases the calories in the diet and the macronutrients for all socioeconomic groups. With respect to micronutrients, the expansion of millet in the diet improves the quantities of iron, zinc, riboflavin and niacin in all groups. It, however, has negative effects on calcium, vitamin C, and vitamin A in all groups. The remaining micronutrients (i.e., thiamine, vitamin B6 and folate) showed differences by group and the reduction of vitamin A might be explained by the partial substitution of matooke in the diet.

Moreover, the implication of the results indicate that under current preferences, substantially increasing the quantity of millet in consumers’ diet will require well targeted incentives to motivate consumers’ appreciation for millet. These findings remind us, as Chera (2017) remarks, that appeals to consumers cannot be a mere afterthought, nor can they simply be framed in terms of development policy or agricultural advantages. The potential nutritional, environmental and economic benefits of embracing agricultural biodiversity are not likely to be enough to change consumers’ preferences for crops such as millets. There is the need rather to bring underutilized crops closer to consumers’ tastes and preferences. This will be an interdisciplinary process, with roles for both natural and social scientists. Only in this way will the millet value chain in Uganda (or similarly, for other orphan crops here and elsewhere) bring sustainable economic, environmental, food security and nutritional benefits.

## Supplementary Information

**Additional file 1**.** Table A1**. Calibrated elasticities - Group rural.** Table A2**. Calibrated elasticities - Group urban-lower quintiles.** Table A3**. Calibrated elasticities - Group urban-upper quintiles.** Table A4**. Food composition (based on 100 grams).

## Data Availability

The data used on the paper is available from the corresponding author upon request.

## Abbreviations

ADBG: African Development Bank Group
AOCC: African Orphan Crops Consortium
ATDF: African Technology Development Forum
FAO: United Nations Food and Agriculture Organization
FAOSTAT: FAO Statistical Office
GNPD: Mintel’s Global New Product Development
ICRISAT: International Crops Research Institute for the Semi-Arid Tropics
LSMS: Living Standard Measurement Survey
MAR: Mean Adequacy Ratio
MER: Mean Excess Ratio
UBOS: Uganda Bureau of Statistics
UNHS: Ugandan National Household Survey
UNPA: Ugandan National Planning Authority
USAID: U.S. Agency for International Development
VBA: Visual Basic for Application
WFP: World Food Programme

## Footnotes

1 This significant decrease happened despite millet being described as a staple food for many communities in different parts of Uganda; besides being grown for food and income, it is considered central to many cultural practices, such as child naming, traditional marriage ceremonies, and welcoming special guests (GlobalFoodMate 2013). Gierend and Orr (2015) indicate that millets in Uganda have experienced a decline in availability and consumption, with the latter apparently reducing from 6 kg/year in 2000 to 5.7 kg/year in 2013. They suggest this was due to a decline in millet cultivation after civil unrest in 2007. Gierend et al. (2014) also mention area cultivated, total production and sector value declines between 1992 and 2012, with a sharp drop in 2008 due to political unrest.

2 As data are in per capita terms (not gender differentiated), we estimated the maximum nutritional requirement for male and female populations (see Omiat and Shively 2007). It should also be noted that, due to the lack of nutritional information, it was not possible to compute the Mean Excess Ratio (MER). This is an indicator of low nutritional quality that is likely important for more affluent population groups and is calculated as the mean daily percentage of maximum recommended values for three harmful nutrients, namely, saturated fats, sodium and free sugars (Vieux et al. 2013).

3 As noted by one of the reviewers because the estimation of the demand model was done using a cross section dataset, there is the possibility that the results (i.e., the elasticities) could be biased due to an endogeneity problem coming from measurement error in prices and household heterogeneity. However, the estimates produced by Boysen (2016) took into consideration both issues. The former problem was controlled applying the Deaton (1997) procedure and the latter by including detailed socioeconomic characteristics into the demand estimation step.

4 Conditional demand figures are not presented here as they are intermediate results required to compute the unconditional elasticities. They are however available from the authors on request.

5 Note that Boysen (2016) only provided relative prices and did not report quantities.

6 $\mathrm{C}\left(\mathrm{p},{\mathrm{p}}_{\mathrm{z}},\uptheta \right)$ is a quasi-expenditure function since it relates to an incomplete demand system (i.e., it represents only a part of total expenditure, such as food expenditure). From here onwards however it is referred to simply as the ‘expenditure function’ for simplicity.

7 According to Mintel’s Global New Product Development (GNPD) database, over 10,000 products sold in South Africa and Nigeria are made from wheat and potato. In contrast, only a few products incorporate underutilized crops. Yet academic research has explored the behaviour of doughs formed from underutilized crop flours (e.g., Angilioni et al. 2013), revealing there is potential for their substitution of staple crop flours.
