# Supplementary material for: Exploring the effects of increasing underutilized crops on consumers’ diets: the case of millet in Uganda
Source: Agric Food Econ. 2022 Jan 5;10(1):1. doi: 10.1186/s40100-021-00206-3 (PMC8752535; doi:10.1186/s40100-021-00206-3)
Supplement: Supplementary file 1 — Additional file 1. Table A1. Calibrated elasticities - Group rural. Table A2. Calibrated elasticities - Group urban-lower quintiles. Table A3. Calibrated elasticities - Group urban-upper quintiles. Table A4. Food composition (based on 100 grams). [file 40100_2021_206_MOESM1_ESM.docx]

**Annex**

Table A1: Calibrated elasticities - Group rural

|  | Millet | Maize | Rice | Bread | Sorgh. | Sweet | Cass. | Matoo. | Veget. | Fruits | Beef | Pork | Goat | Chick. | Other | Eggs | Fish | Puls., | Milk | Oils | Sugar | Soda | Other | Coffee | Food | Beer | Other | Other |
| --- | --- | --- | --- | --- | --- | --- | --- | --- | --- | --- | --- | --- | --- | --- | --- | --- | --- | --- | --- | --- | --- | --- | --- | --- | --- | --- | --- | --- |
|  |  |  |  |  |  | Irish |  |  |  |  |  |  | meat |  | meat |  |  | leg., |  | and |  |  | juices | and | away |  | alcoh. | foods |
|  |  |  |  |  |  | Potat. |  |  |  |  |  |  |  |  |  |  |  | nuts |  | fats |  |  |  | tea | home |  | bev. |  |
|  |  |  |  |  |  |  |  |  |  |  |  |  |  |  |  |  |  |  |  |  |  |  |  |  |  |  |  |  |
| **Hicksian elasticities** |  |  |  |  |  |  |  |  |  |  |  |  |  |  |  |  |  |  |  |  |  |  |  |  |  |  |  |  |
| Millet | -1.219 | 0.021 | 0.003 | 0.002 | 0.005 | 0.023 | 0.014 | 0.027 | 0.001 | 0.008 | 0.014 | 0.004 | 0.005 | 0.009 | 0.001 | 0.002 | 0.013 | 0.016 | 0.009 | 0.003 | 0.008 | 0.001 | 0.000 | 0.000 | 0.006 | 0.001 | 0.003 | 0.004 |
| Maize | 0.003 | -1.391 | 0.003 | 0.002 | 0.005 | 0.024 | 0.014 | 0.029 | 0.001 | 0.009 | 0.015 | 0.004 | 0.005 | 0.010 | 0.001 | 0.002 | 0.014 | 0.017 | 0.010 | 0.003 | 0.008 | 0.001 | 0.000 | 0.000 | 0.006 | 0.001 | 0.003 | 0.004 |
| Rice | 0.003 | 0.021 | -1.198 | 0.002 | 0.005 | 0.023 | 0.014 | 0.027 | 0.001 | 0.008 | 0.014 | 0.004 | 0.005 | 0.009 | 0.001 | 0.002 | 0.013 | 0.016 | 0.009 | 0.003 | 0.008 | 0.001 | 0.000 | 0.000 | 0.006 | 0.001 | 0.003 | 0.004 |
| Bread | 0.003 | 0.021 | 0.003 | -1.228 | 0.005 | 0.023 | 0.014 | 0.027 | 0.001 | 0.008 | 0.014 | 0.004 | 0.005 | 0.009 | 0.001 | 0.002 | 0.013 | 0.016 | 0.009 | 0.003 | 0.008 | 0.001 | 0.000 | 0.000 | 0.006 | 0.001 | 0.003 | 0.004 |
| Sorghum | 0.003 | 0.021 | 0.003 | 0.002 | -1.305 | 0.023 | 0.014 | 0.027 | 0.001 | 0.008 | 0.014 | 0.004 | 0.005 | 0.009 | 0.001 | 0.002 | 0.013 | 0.016 | 0.009 | 0.003 | 0.008 | 0.001 | 0.000 | 0.000 | 0.006 | 0.001 | 0.003 | 0.004 |
| Sweet and Irish Potatoes | 0.003 | 0.023 | 0.004 | 0.002 | 0.005 | -0.926 | 0.015 | 0.030 | 0.001 | 0.009 | 0.015 | 0.004 | 0.005 | 0.010 | 0.001 | 0.002 | 0.014 | 0.017 | 0.010 | 0.004 | 0.008 | 0.001 | 0.000 | 0.000 | 0.006 | 0.001 | 0.004 | 0.004 |
| Cassava | 0.002 | 0.012 | 0.002 | 0.001 | 0.003 | 0.013 | -0.532 | 0.016 | 0.000 | 0.005 | 0.008 | 0.002 | 0.003 | 0.005 | 0.000 | 0.001 | 0.008 | 0.009 | 0.005 | 0.002 | 0.004 | 0.001 | 0.000 | 0.000 | 0.003 | 0.001 | 0.002 | 0.002 |
| Matooke | 0.005 | 0.034 | 0.005 | 0.002 | 0.008 | 0.037 | 0.022 | -0.873 | 0.001 | 0.014 | 0.022 | 0.006 | 0.007 | 0.015 | 0.001 | 0.003 | 0.022 | 0.025 | 0.015 | 0.005 | 0.013 | 0.002 | 0.000 | 0.001 | 0.010 | 0.001 | 0.005 | 0.006 |
| Vegetables | 0.000 | 0.001 | 0.000 | 0.000 | 0.000 | 0.001 | 0.001 | 0.001 | -0.468 | 0.000 | 0.001 | 0.000 | 0.000 | 0.000 | 0.000 | 0.000 | 0.001 | 0.001 | 0.000 | 0.000 | 0.000 | 0.000 | 0.000 | 0.000 | 0.000 | 0.000 | 0.000 | 0.000 |
| Fruits | 0.004 | 0.027 | 0.004 | 0.002 | 0.006 | 0.030 | 0.018 | 0.036 | 0.001 | -1.186 | 0.018 | 0.005 | 0.006 | 0.012 | 0.001 | 0.002 | 0.017 | 0.021 | 0.012 | 0.004 | 0.010 | 0.002 | 0.000 | 0.001 | 0.008 | 0.001 | 0.004 | 0.005 |
| Beef | 0.006 | 0.042 | 0.007 | 0.003 | 0.010 | 0.046 | 0.027 | 0.055 | 0.001 | 0.017 | -1.043 | 0.008 | 0.009 | 0.019 | 0.002 | 0.003 | 0.027 | 0.032 | 0.019 | 0.007 | 0.016 | 0.002 | 0.000 | 0.001 | 0.012 | 0.002 | 0.007 | 0.008 |
| Pork | 0.006 | 0.042 | 0.007 | 0.003 | 0.010 | 0.047 | 0.028 | 0.055 | 0.001 | 0.017 | 0.028 | -1.103 | 0.009 | 0.019 | 0.002 | 0.003 | 0.027 | 0.032 | 0.019 | 0.007 | 0.016 | 0.002 | 0.000 | 0.001 | 0.012 | 0.002 | 0.007 | 0.008 |
| Goat meat | 0.006 | 0.042 | 0.007 | 0.003 | 0.010 | 0.047 | 0.028 | 0.055 | 0.001 | 0.017 | 0.028 | 0.008 | -1.093 | 0.019 | 0.002 | 0.003 | 0.027 | 0.032 | 0.019 | 0.007 | 0.016 | 0.002 | 0.000 | 0.001 | 0.012 | 0.002 | 0.007 | 0.008 |
| Chicken | 0.006 | 0.042 | 0.007 | 0.003 | 0.010 | 0.047 | 0.028 | 0.055 | 0.001 | 0.017 | 0.028 | 0.008 | 0.009 | -1.096 | 0.002 | 0.003 | 0.027 | 0.032 | 0.019 | 0.007 | 0.016 | 0.002 | 0.000 | 0.001 | 0.012 | 0.002 | 0.007 | 0.008 |
| Other meat | 0.006 | 0.042 | 0.007 | 0.003 | 0.010 | 0.046 | 0.027 | 0.055 | 0.001 | 0.017 | 0.028 | 0.008 | 0.009 | 0.019 | -1.117 | 0.003 | 0.027 | 0.032 | 0.019 | 0.007 | 0.016 | 0.002 | 0.000 | 0.001 | 0.012 | 0.002 | 0.007 | 0.008 |
| Eggs | 0.006 | 0.043 | 0.007 | 0.003 | 0.010 | 0.047 | 0.028 | 0.056 | 0.001 | 0.017 | 0.028 | 0.008 | 0.009 | 0.019 | 0.002 | -1.135 | 0.027 | 0.032 | 0.019 | 0.007 | 0.016 | 0.002 | 0.000 | 0.001 | 0.012 | 0.002 | 0.007 | 0.008 |
| Fish | 0.005 | 0.036 | 0.006 | 0.003 | 0.008 | 0.039 | 0.023 | 0.047 | 0.001 | 0.014 | 0.024 | 0.007 | 0.008 | 0.016 | 0.001 | 0.003 | -1.333 | 0.027 | 0.016 | 0.006 | 0.013 | 0.002 | 0.000 | 0.001 | 0.010 | 0.001 | 0.006 | 0.007 |
| Pulses, legumes, nuts | 0.002 | 0.015 | 0.002 | 0.001 | 0.003 | 0.016 | 0.009 | 0.019 | 0.000 | 0.006 | 0.010 | 0.003 | 0.003 | 0.006 | 0.001 | 0.001 | 0.009 | -0.716 | 0.007 | 0.002 | 0.005 | 0.001 | 0.000 | 0.000 | 0.004 | 0.001 | 0.002 | 0.003 |
| Milk | 0.005 | 0.034 | 0.005 | 0.002 | 0.008 | 0.038 | 0.022 | 0.045 | 0.001 | 0.014 | 0.023 | 0.006 | 0.008 | 0.015 | 0.001 | 0.003 | 0.022 | 0.026 | -1.393 | 0.005 | 0.013 | 0.002 | 0.000 | 0.001 | 0.010 | 0.001 | 0.005 | 0.006 |
| Oils and fats | 0.002 | 0.017 | 0.003 | 0.001 | 0.004 | 0.019 | 0.011 | 0.022 | 0.001 | 0.007 | 0.011 | 0.003 | 0.004 | 0.008 | 0.001 | 0.001 | 0.011 | 0.013 | 0.008 | -0.601 | 0.006 | 0.001 | 0.000 | 0.000 | 0.005 | 0.001 | 0.003 | 0.003 |
| Sugar | 0.004 | 0.026 | 0.004 | 0.002 | 0.006 | 0.028 | 0.017 | 0.034 | 0.001 | 0.010 | 0.017 | 0.005 | 0.006 | 0.011 | 0.001 | 0.002 | 0.016 | 0.020 | 0.012 | 0.004 | -0.924 | 0.001 | 0.000 | 0.001 | 0.007 | 0.001 | 0.004 | 0.005 |
| Soda | 0.004 | 0.026 | 0.004 | 0.002 | 0.006 | 0.028 | 0.017 | 0.034 | 0.001 | 0.010 | 0.017 | 0.005 | 0.006 | 0.011 | 0.001 | 0.002 | 0.016 | 0.019 | 0.012 | 0.004 | 0.010 | -0.961 | 0.000 | 0.001 | 0.007 | 0.001 | 0.004 | 0.005 |
| Other juices | 0.004 | 0.026 | 0.004 | 0.002 | 0.006 | 0.028 | 0.017 | 0.034 | 0.001 | 0.010 | 0.017 | 0.005 | 0.006 | 0.011 | 0.001 | 0.002 | 0.017 | 0.020 | 0.012 | 0.004 | 0.010 | 0.001 | -0.818 | 0.001 | 0.007 | 0.001 | 0.004 | 0.005 |
| Coffee & tea | 0.003 | 0.024 | 0.004 | 0.002 | 0.006 | 0.026 | 0.015 | 0.031 | 0.001 | 0.010 | 0.016 | 0.004 | 0.005 | 0.011 | 0.001 | 0.002 | 0.015 | 0.018 | 0.011 | 0.004 | 0.009 | 0.001 | 0.000 | -0.946 | 0.007 | 0.001 | 0.004 | 0.004 |
| Food away | 0.003 | 0.024 | 0.004 | 0.002 | 0.006 | 0.026 | 0.015 | 0.031 | 0.001 | 0.010 | 0.016 | 0.004 | 0.005 | 0.011 | 0.001 | 0.002 | 0.015 | 0.018 | 0.011 | 0.004 | 0.009 | 0.001 | 0.000 | 0.000 | -1.549 | 0.001 | 0.004 | 0.004 |
| Beer | 0.003 | 0.024 | 0.004 | 0.002 | 0.006 | 0.026 | 0.015 | 0.031 | 0.001 | 0.010 | 0.016 | 0.004 | 0.005 | 0.011 | 0.001 | 0.002 | 0.015 | 0.018 | 0.011 | 0.004 | 0.009 | 0.001 | 0.000 | 0.000 | 0.007 | -0.680 | 0.004 | 0.004 |
| Other alcoholic beverages | 0.003 | 0.024 | 0.004 | 0.002 | 0.006 | 0.026 | 0.015 | 0.031 | 0.001 | 0.010 | 0.016 | 0.004 | 0.005 | 0.011 | 0.001 | 0.002 | 0.015 | 0.018 | 0.011 | 0.004 | 0.009 | 0.001 | 0.000 | 0.000 | 0.007 | 0.001 | -1.793 | 0.004 |
| Other foods | 0.003 | 0.024 | 0.004 | 0.002 | 0.006 | 0.026 | 0.015 | 0.031 | 0.001 | 0.010 | 0.016 | 0.004 | 0.005 | 0.011 | 0.001 | 0.002 | 0.015 | 0.018 | 0.011 | 0.004 | 0.009 | 0.001 | 0.000 | 0.000 | 0.007 | 0.001 | 0.004 | -1.337 |
| **Marshallian elasticities** |  |  |  |  |  |  |  |  |  |  |  |  |  |  |  |  |  |  |  |  |  |  |  |  |  |  |  |  |
| Millet | -1.228 | -0.034 | -0.006 | -0.003 | -0.009 | -0.036 | -0.052 | -0.020 | -0.036 | -0.010 | -0.005 | -0.001 | -0.002 | -0.004 | 0.000 | -0.001 | -0.009 | -0.048 | -0.007 | -0.008 | -0.010 | -0.002 | 0.000 | -0.001 | -0.009 | -0.001 | -0.005 | -0.006 |
| Maize | -0.006 | -1.450 | -0.006 | -0.003 | -0.010 | -0.039 | -0.056 | -0.022 | -0.039 | -0.010 | -0.006 | -0.002 | -0.002 | -0.004 | 0.000 | -0.001 | -0.009 | -0.051 | -0.007 | -0.009 | -0.011 | -0.002 | 0.000 | -0.001 | -0.009 | -0.001 | -0.005 | -0.006 |
| Rice | -0.006 | -0.034 | -1.207 | -0.003 | -0.009 | -0.036 | -0.052 | -0.020 | -0.036 | -0.010 | -0.005 | -0.001 | -0.002 | -0.004 | 0.000 | -0.001 | -0.009 | -0.048 | -0.007 | -0.008 | -0.010 | -0.002 | 0.000 | -0.001 | -0.009 | -0.001 | -0.005 | -0.006 |
| Bread | -0.006 | -0.034 | -0.006 | -1.233 | -0.009 | -0.037 | -0.052 | -0.020 | -0.036 | -0.010 | -0.005 | -0.001 | -0.002 | -0.004 | 0.000 | -0.001 | -0.009 | -0.048 | -0.007 | -0.008 | -0.010 | -0.002 | 0.000 | -0.001 | -0.009 | -0.001 | -0.005 | -0.006 |
| Sorghum | -0.006 | -0.034 | -0.006 | -0.003 | -1.319 | -0.036 | -0.052 | -0.020 | -0.036 | -0.010 | -0.005 | -0.001 | -0.002 | -0.004 | 0.000 | -0.001 | -0.009 | -0.048 | -0.007 | -0.008 | -0.010 | -0.002 | 0.000 | -0.001 | -0.009 | -0.001 | -0.005 | -0.006 |
| Sweet and Irish Potatoes | -0.006 | -0.037 | -0.006 | -0.003 | -0.010 | -0.990 | -0.057 | -0.022 | -0.039 | -0.011 | -0.006 | -0.002 | -0.002 | -0.004 | 0.000 | -0.001 | -0.009 | -0.052 | -0.007 | -0.009 | -0.011 | -0.002 | 0.000 | -0.001 | -0.009 | -0.001 | -0.005 | -0.006 |
| Cassava | -0.003 | -0.020 | -0.003 | -0.002 | -0.005 | -0.021 | -0.570 | -0.012 | -0.021 | -0.006 | -0.003 | -0.001 | -0.001 | -0.002 | 0.000 | 0.000 | -0.005 | -0.028 | -0.004 | -0.005 | -0.006 | -0.001 | 0.000 | 0.000 | -0.005 | -0.001 | -0.003 | -0.003 |
| Matooke | -0.009 | -0.055 | -0.009 | -0.004 | -0.014 | -0.059 | -0.084 | -0.950 | -0.058 | -0.016 | -0.009 | -0.002 | -0.003 | -0.006 | 0.000 | -0.001 | -0.014 | -0.077 | -0.011 | -0.013 | -0.016 | -0.002 | 0.000 | -0.001 | -0.014 | -0.002 | -0.008 | -0.009 |
| Vegetables | 0.000 | -0.002 | 0.000 | 0.000 | 0.000 | -0.002 | -0.003 | -0.001 | -0.470 | -0.001 | 0.000 | 0.000 | 0.000 | 0.000 | 0.000 | 0.000 | 0.000 | -0.003 | 0.000 | 0.000 | -0.001 | 0.000 | 0.000 | 0.000 | 0.000 | 0.000 | 0.000 | 0.000 |
| Fruits | -0.007 | -0.045 | -0.008 | -0.004 | -0.012 | -0.048 | -0.068 | -0.026 | -0.047 | -1.210 | -0.007 | -0.002 | -0.002 | -0.005 | 0.000 | -0.001 | -0.011 | -0.063 | -0.009 | -0.010 | -0.013 | -0.002 | 0.000 | -0.001 | -0.011 | -0.002 | -0.006 | -0.007 |
| Beef | -0.011 | -0.069 | -0.012 | -0.006 | -0.018 | -0.074 | -0.106 | -0.041 | -0.073 | -0.020 | -1.082 | -0.003 | -0.004 | -0.007 | -0.001 | -0.001 | -0.017 | -0.097 | -0.014 | -0.016 | -0.020 | -0.003 | 0.000 | -0.001 | -0.018 | -0.003 | -0.010 | -0.012 |
| Pork | -0.011 | -0.069 | -0.012 | -0.006 | -0.018 | -0.074 | -0.106 | -0.041 | -0.073 | -0.020 | -0.011 | -1.114 | -0.004 | -0.007 | -0.001 | -0.001 | -0.017 | -0.097 | -0.014 | -0.016 | -0.020 | -0.003 | 0.000 | -0.001 | -0.018 | -0.003 | -0.010 | -0.012 |
| Goat meat | -0.011 | -0.069 | -0.012 | -0.006 | -0.018 | -0.074 | -0.106 | -0.041 | -0.073 | -0.020 | -0.011 | -0.003 | -1.106 | -0.007 | -0.001 | -0.001 | -0.017 | -0.097 | -0.014 | -0.016 | -0.020 | -0.003 | 0.000 | -0.001 | -0.018 | -0.003 | -0.010 | -0.012 |
| Chicken | -0.011 | -0.069 | -0.012 | -0.006 | -0.018 | -0.074 | -0.106 | -0.041 | -0.073 | -0.020 | -0.011 | -0.003 | -0.004 | -1.122 | -0.001 | -0.001 | -0.017 | -0.097 | -0.014 | -0.016 | -0.020 | -0.003 | 0.000 | -0.001 | -0.018 | -0.003 | -0.010 | -0.012 |
| Other meat | -0.011 | -0.069 | -0.012 | -0.006 | -0.018 | -0.074 | -0.105 | -0.041 | -0.073 | -0.020 | -0.011 | -0.003 | -0.004 | -0.007 | -1.119 | -0.001 | -0.017 | -0.097 | -0.014 | -0.016 | -0.020 | -0.003 | 0.000 | -0.001 | -0.018 | -0.003 | -0.010 | -0.012 |
| Eggs | -0.011 | -0.070 | -0.012 | -0.006 | -0.018 | -0.074 | -0.106 | -0.041 | -0.074 | -0.020 | -0.011 | -0.003 | -0.004 | -0.007 | -0.001 | -1.139 | -0.018 | -0.098 | -0.014 | -0.016 | -0.020 | -0.003 | 0.000 | -0.001 | -0.018 | -0.003 | -0.010 | -0.012 |
| Fish | -0.009 | -0.058 | -0.010 | -0.005 | -0.015 | -0.062 | -0.089 | -0.035 | -0.062 | -0.017 | -0.009 | -0.003 | -0.003 | -0.006 | -0.001 | -0.001 | -1.370 | -0.082 | -0.012 | -0.014 | -0.017 | -0.003 | 0.000 | -0.001 | -0.015 | -0.002 | -0.008 | -0.010 |
| Pulses, legumes, nuts | -0.004 | -0.024 | -0.004 | -0.002 | -0.006 | -0.025 | -0.036 | -0.014 | -0.025 | -0.007 | -0.004 | -0.001 | -0.001 | -0.002 | 0.000 | 0.000 | -0.006 | -0.760 | -0.005 | -0.006 | -0.007 | -0.001 | 0.000 | 0.000 | -0.006 | -0.001 | -0.003 | -0.004 |
| Milk | -0.009 | -0.056 | -0.010 | -0.004 | -0.015 | -0.059 | -0.085 | -0.033 | -0.059 | -0.016 | -0.009 | -0.002 | -0.003 | -0.006 | 0.000 | -0.001 | -0.014 | -0.078 | -1.420 | -0.013 | -0.016 | -0.002 | 0.000 | -0.001 | -0.014 | -0.002 | -0.008 | -0.009 |
| Oils and fats | -0.004 | -0.028 | -0.005 | -0.002 | -0.007 | -0.030 | -0.042 | -0.016 | -0.029 | -0.008 | -0.004 | -0.001 | -0.001 | -0.003 | 0.000 | 0.000 | -0.007 | -0.039 | -0.006 | -0.610 | -0.008 | -0.001 | 0.000 | -0.001 | -0.007 | -0.001 | -0.004 | -0.005 |
| Sugar | -0.007 | -0.042 | -0.007 | -0.003 | -0.011 | -0.045 | -0.064 | -0.025 | -0.045 | -0.012 | -0.007 | -0.002 | -0.002 | -0.004 | 0.000 | -0.001 | -0.011 | -0.059 | -0.008 | -0.010 | -0.946 | -0.002 | 0.000 | -0.001 | -0.011 | -0.002 | -0.006 | -0.007 |
| Soda | -0.007 | -0.042 | -0.007 | -0.003 | -0.011 | -0.045 | -0.064 | -0.025 | -0.045 | -0.012 | -0.007 | -0.002 | -0.002 | -0.004 | 0.000 | -0.001 | -0.011 | -0.059 | -0.008 | -0.010 | -0.012 | -0.965 | 0.000 | -0.001 | -0.011 | -0.002 | -0.006 | -0.007 |
| Other juices | -0.007 | -0.042 | -0.007 | -0.003 | -0.011 | -0.045 | -0.065 | -0.025 | -0.045 | -0.012 | -0.007 | -0.002 | -0.002 | -0.004 | 0.000 | -0.001 | -0.011 | -0.059 | -0.008 | -0.010 | -0.012 | -0.002 | -0.818 | -0.001 | -0.011 | -0.002 | -0.006 | -0.007 |
| Coffee & tea | -0.006 | -0.039 | -0.007 | -0.003 | -0.010 | -0.041 | -0.059 | -0.023 | -0.041 | -0.011 | -0.006 | -0.002 | -0.002 | -0.004 | 0.000 | -0.001 | -0.010 | -0.054 | -0.008 | -0.009 | -0.011 | -0.002 | 0.000 | -0.947 | -0.010 | -0.001 | -0.005 | -0.007 |
| Food away | -0.006 | -0.039 | -0.007 | -0.003 | -0.010 | -0.042 | -0.060 | -0.023 | -0.041 | -0.011 | -0.006 | -0.002 | -0.002 | -0.004 | 0.000 | -0.001 | -0.010 | -0.055 | -0.008 | -0.009 | -0.011 | -0.002 | 0.000 | -0.001 | -1.565 | -0.001 | -0.005 | -0.007 |
| Beer | -0.006 | -0.039 | -0.007 | -0.003 | -0.010 | -0.041 | -0.059 | -0.023 | -0.041 | -0.011 | -0.006 | -0.002 | -0.002 | -0.004 | 0.000 | -0.001 | -0.010 | -0.055 | -0.008 | -0.009 | -0.011 | -0.002 | 0.000 | -0.001 | -0.010 | -0.683 | -0.005 | -0.007 |
| Other alcoholic beverages | -0.006 | -0.039 | -0.007 | -0.003 | -0.010 | -0.041 | -0.059 | -0.023 | -0.041 | -0.011 | -0.006 | -0.002 | -0.002 | -0.004 | 0.000 | -0.001 | -0.010 | -0.055 | -0.008 | -0.009 | -0.011 | -0.002 | 0.000 | -0.001 | -0.010 | -0.001 | -1.802 | -0.007 |
| Other foods | -0.006 | -0.039 | -0.007 | -0.003 | -0.010 | -0.041 | -0.059 | -0.023 | -0.041 | -0.011 | -0.006 | -0.002 | -0.002 | -0.004 | 0.000 | -0.001 | -0.010 | -0.054 | -0.008 | -0.009 | -0.011 | -0.002 | 0.000 | -0.001 | -0.010 | -0.001 | -0.005 | -1.348 |
| **Income elasticities** | 0.950 | 1.010 | 0.949 | 0.951 | 0.950 | 1.030 | 0.550 | 1.530 | 0.050 | 1.240 | 1.919 | 1.920 | 1.921 | 1.920 | 1.917 | 1.934 | 1.620 | 0.660 | 1.550 | 0.770 | 1.170 | 1.170 | 1.174 | 1.077 | 1.081 | 1.078 | 1.080 | 1.078 |
|  |  |  |  |  |  |  |  |  |  |  |  |  |  |  |  |  |  |  |  |  |  |  |  |  |  |  |  |  |

Table A2: Calibrated elasticities - Group urban-lower quintiles

|  | Millet | Maize | Rice | Bread | Sorgh. | Sweet | Cass. | Matoo. | Veget. | Fruits | Beef | Pork | Goat | Chick. | Other | Eggs | Fish | Puls., | Milk | Oils | Sugar | Soda | Other | Coffee | Food | Beer | Other | Other |
| --- | --- | --- | --- | --- | --- | --- | --- | --- | --- | --- | --- | --- | --- | --- | --- | --- | --- | --- | --- | --- | --- | --- | --- | --- | --- | --- | --- | --- |
|  |  |  |  |  |  | Irish |  |  |  |  |  |  | meat |  | meat |  |  | leg., |  | and |  |  | juices | and | away |  | alcoh. | foods |
|  |  |  |  |  |  | Potat. |  |  |  |  |  |  |  |  |  |  |  | nuts |  | fats |  |  |  | tea | home |  | bev. |  |
|  |  |  |  |  |  |  |  |  |  |  |  |  |  |  |  |  |  |  |  |  |  |  |  |  |  |  |  |  |
| **Hicksian elasticities** |  |  |  |  |  |  |  |  |  |  |  |  |  |  |  |  |  |  |  |  |  |  |  |  |  |  |  |  |
| Millet | -1.007 | 0.010 | 0.003 | 0.002 | 0.001 | 0.011 | 0.006 | 0.016 | 0.005 | 0.006 | 0.013 | 0.003 | 0.003 | 0.005 | 0.000 | 0.001 | 0.010 | 0.011 | 0.007 | 0.003 | 0.005 | 0.001 | 0.000 | 0.001 | 0.009 | 0.001 | 0.001 | 0.004 |
| Maize | 0.001 | -1.392 | 0.004 | 0.002 | 0.002 | 0.013 | 0.008 | 0.019 | 0.005 | 0.007 | 0.015 | 0.003 | 0.003 | 0.006 | 0.000 | 0.002 | 0.012 | 0.013 | 0.009 | 0.004 | 0.007 | 0.001 | 0.000 | 0.001 | 0.011 | 0.001 | 0.001 | 0.005 |
| Rice | 0.001 | 0.010 | -0.965 | 0.002 | 0.001 | 0.011 | 0.006 | 0.016 | 0.005 | 0.006 | 0.013 | 0.003 | 0.003 | 0.005 | 0.000 | 0.001 | 0.010 | 0.011 | 0.007 | 0.003 | 0.005 | 0.001 | 0.000 | 0.001 | 0.009 | 0.001 | 0.001 | 0.004 |
| Bread | 0.001 | 0.010 | 0.003 | -1.187 | 0.001 | 0.011 | 0.007 | 0.016 | 0.005 | 0.006 | 0.013 | 0.003 | 0.003 | 0.005 | 0.000 | 0.001 | 0.010 | 0.011 | 0.008 | 0.003 | 0.005 | 0.001 | 0.000 | 0.001 | 0.009 | 0.001 | 0.001 | 0.004 |
| Sorghum | 0.001 | 0.010 | 0.003 | 0.002 | -0.988 | 0.011 | 0.006 | 0.016 | 0.005 | 0.006 | 0.013 | 0.003 | 0.003 | 0.005 | 0.000 | 0.001 | 0.010 | 0.011 | 0.007 | 0.003 | 0.005 | 0.001 | 0.000 | 0.001 | 0.009 | 0.001 | 0.001 | 0.004 |
| Sweet and Irish Potatoes | 0.002 | 0.014 | 0.004 | 0.002 | 0.002 | -0.666 | 0.008 | 0.021 | 0.006 | 0.007 | 0.016 | 0.003 | 0.003 | 0.006 | 0.000 | 0.002 | 0.013 | 0.014 | 0.010 | 0.004 | 0.007 | 0.001 | 0.000 | 0.001 | 0.012 | 0.001 | 0.001 | 0.005 |
| Cassava | 0.001 | 0.011 | 0.003 | 0.002 | 0.001 | 0.011 | -0.668 | 0.016 | 0.005 | 0.006 | 0.013 | 0.003 | 0.003 | 0.005 | 0.000 | 0.002 | 0.010 | 0.011 | 0.008 | 0.003 | 0.006 | 0.001 | 0.000 | 0.001 | 0.009 | 0.001 | 0.001 | 0.004 |
| Matooke | 0.003 | 0.022 | 0.007 | 0.004 | 0.003 | 0.024 | 0.014 | -1.210 | 0.010 | 0.012 | 0.027 | 0.006 | 0.006 | 0.010 | 0.001 | 0.003 | 0.021 | 0.024 | 0.016 | 0.007 | 0.012 | 0.001 | 0.000 | 0.001 | 0.019 | 0.001 | 0.002 | 0.008 |
| Vegetables | 0.001 | 0.007 | 0.002 | 0.001 | 0.001 | 0.008 | 0.005 | 0.011 | -0.622 | 0.004 | 0.009 | 0.002 | 0.002 | 0.003 | 0.000 | 0.001 | 0.007 | 0.008 | 0.005 | 0.002 | 0.004 | 0.001 | 0.000 | 0.000 | 0.007 | 0.000 | 0.001 | 0.003 |
| Fruits | 0.002 | 0.019 | 0.006 | 0.003 | 0.002 | 0.020 | 0.012 | 0.029 | 0.008 | -0.615 | 0.023 | 0.005 | 0.005 | 0.009 | 0.001 | 0.003 | 0.018 | 0.020 | 0.013 | 0.006 | 0.010 | 0.001 | 0.000 | 0.001 | 0.016 | 0.001 | 0.002 | 0.007 |
| Beef | 0.004 | 0.033 | 0.010 | 0.006 | 0.004 | 0.035 | 0.020 | 0.050 | 0.014 | 0.018 | -0.839 | 0.008 | 0.008 | 0.015 | 0.001 | 0.005 | 0.031 | 0.035 | 0.023 | 0.010 | 0.017 | 0.002 | 0.001 | 0.002 | 0.029 | 0.002 | 0.003 | 0.012 |
| Pork | 0.004 | 0.033 | 0.010 | 0.006 | 0.004 | 0.035 | 0.020 | 0.050 | 0.014 | 0.018 | 0.040 | -0.907 | 0.008 | 0.015 | 0.001 | 0.005 | 0.031 | 0.035 | 0.023 | 0.010 | 0.017 | 0.002 | 0.001 | 0.002 | 0.029 | 0.002 | 0.003 | 0.012 |
| Goat meat | 0.004 | 0.033 | 0.010 | 0.006 | 0.004 | 0.035 | 0.020 | 0.050 | 0.014 | 0.018 | 0.040 | 0.008 | -0.903 | 0.015 | 0.001 | 0.005 | 0.031 | 0.035 | 0.024 | 0.010 | 0.017 | 0.002 | 0.001 | 0.002 | 0.029 | 0.002 | 0.003 | 0.012 |
| Chicken | 0.004 | 0.033 | 0.010 | 0.006 | 0.004 | 0.035 | 0.020 | 0.050 | 0.014 | 0.018 | 0.040 | 0.008 | 0.008 | -0.857 | 0.001 | 0.005 | 0.031 | 0.035 | 0.023 | 0.010 | 0.017 | 0.002 | 0.001 | 0.002 | 0.029 | 0.002 | 0.003 | 0.012 |
| Other meat | 0.004 | 0.033 | 0.010 | 0.006 | 0.004 | 0.035 | 0.020 | 0.050 | 0.014 | 0.018 | 0.040 | 0.008 | 0.008 | 0.015 | -0.889 | 0.005 | 0.031 | 0.035 | 0.023 | 0.010 | 0.017 | 0.002 | 0.001 | 0.002 | 0.028 | 0.002 | 0.003 | 0.012 |
| Eggs | 0.004 | 0.033 | 0.011 | 0.006 | 0.004 | 0.035 | 0.020 | 0.050 | 0.014 | 0.018 | 0.040 | 0.008 | 0.008 | 0.015 | 0.001 | -0.989 | 0.032 | 0.035 | 0.024 | 0.010 | 0.017 | 0.002 | 0.001 | 0.002 | 0.029 | 0.002 | 0.003 | 0.012 |
| Fish | 0.003 | 0.026 | 0.008 | 0.005 | 0.003 | 0.028 | 0.016 | 0.041 | 0.011 | 0.014 | 0.032 | 0.007 | 0.007 | 0.012 | 0.001 | 0.004 | -1.297 | 0.028 | 0.019 | 0.008 | 0.014 | 0.002 | 0.000 | 0.002 | 0.023 | 0.002 | 0.003 | 0.010 |
| Pulses, legumes, nuts | 0.001 | 0.011 | 0.003 | 0.002 | 0.001 | 0.011 | 0.007 | 0.017 | 0.005 | 0.006 | 0.013 | 0.003 | 0.003 | 0.005 | 0.000 | 0.002 | 0.010 | -0.800 | 0.008 | 0.003 | 0.006 | 0.001 | 0.000 | 0.001 | 0.009 | 0.001 | 0.001 | 0.004 |
| Milk | 0.003 | 0.024 | 0.008 | 0.004 | 0.003 | 0.026 | 0.015 | 0.037 | 0.011 | 0.013 | 0.029 | 0.006 | 0.006 | 0.011 | 0.001 | 0.003 | 0.023 | 0.026 | -1.375 | 0.007 | 0.013 | 0.002 | 0.000 | 0.002 | 0.021 | 0.001 | 0.003 | 0.009 |
| Oils and fats | 0.001 | 0.013 | 0.004 | 0.002 | 0.002 | 0.013 | 0.008 | 0.019 | 0.005 | 0.007 | 0.015 | 0.003 | 0.003 | 0.006 | 0.000 | 0.002 | 0.012 | 0.013 | 0.009 | -0.663 | 0.007 | 0.001 | 0.000 | 0.001 | 0.011 | 0.001 | 0.001 | 0.005 |
| Sugar | 0.002 | 0.013 | 0.004 | 0.002 | 0.002 | 0.014 | 0.008 | 0.020 | 0.006 | 0.007 | 0.016 | 0.003 | 0.003 | 0.006 | 0.000 | 0.002 | 0.013 | 0.014 | 0.009 | 0.004 | -1.020 | 0.001 | 0.000 | 0.001 | 0.011 | 0.001 | 0.001 | 0.005 |
| Soda | 0.002 | 0.013 | 0.004 | 0.002 | 0.002 | 0.014 | 0.008 | 0.020 | 0.006 | 0.007 | 0.016 | 0.003 | 0.003 | 0.006 | 0.000 | 0.002 | 0.013 | 0.014 | 0.009 | 0.004 | 0.007 | -0.970 | 0.000 | 0.001 | 0.011 | 0.001 | 0.001 | 0.005 |
| Other juices | 0.002 | 0.013 | 0.004 | 0.002 | 0.002 | 0.014 | 0.008 | 0.020 | 0.006 | 0.007 | 0.016 | 0.003 | 0.003 | 0.006 | 0.000 | 0.002 | 0.013 | 0.014 | 0.009 | 0.004 | 0.007 | 0.001 | -0.982 | 0.001 | 0.011 | 0.001 | 0.001 | 0.005 |
| Coffee & tea | 0.003 | 0.024 | 0.008 | 0.004 | 0.003 | 0.025 | 0.015 | 0.037 | 0.010 | 0.013 | 0.029 | 0.006 | 0.006 | 0.011 | 0.001 | 0.003 | 0.023 | 0.025 | 0.017 | 0.007 | 0.012 | 0.002 | 0.000 | -0.925 | 0.021 | 0.001 | 0.002 | 0.009 |
| Food away | 0.003 | 0.024 | 0.008 | 0.004 | 0.003 | 0.025 | 0.015 | 0.037 | 0.010 | 0.013 | 0.029 | 0.006 | 0.006 | 0.011 | 0.001 | 0.003 | 0.023 | 0.025 | 0.017 | 0.007 | 0.013 | 0.002 | 0.000 | 0.002 | -0.857 | 0.001 | 0.002 | 0.009 |
| Beer | 0.003 | 0.024 | 0.008 | 0.004 | 0.003 | 0.025 | 0.015 | 0.036 | 0.010 | 0.013 | 0.029 | 0.006 | 0.006 | 0.011 | 0.001 | 0.003 | 0.023 | 0.025 | 0.017 | 0.007 | 0.012 | 0.002 | 0.000 | 0.002 | 0.021 | -0.731 | 0.002 | 0.009 |
| Other alcoholic beverages | 0.003 | 0.024 | 0.008 | 0.004 | 0.003 | 0.025 | 0.015 | 0.037 | 0.010 | 0.013 | 0.029 | 0.006 | 0.006 | 0.011 | 0.001 | 0.003 | 0.023 | 0.025 | 0.017 | 0.007 | 0.013 | 0.002 | 0.000 | 0.002 | 0.021 | 0.001 | -2.650 | 0.009 |
| Other foods | 0.003 | 0.024 | 0.008 | 0.004 | 0.003 | 0.025 | 0.015 | 0.037 | 0.010 | 0.013 | 0.029 | 0.006 | 0.006 | 0.011 | 0.001 | 0.003 | 0.023 | 0.025 | 0.017 | 0.007 | 0.012 | 0.002 | 0.000 | 0.002 | 0.021 | 0.001 | 0.002 | -0.933 |
| **Marshallian elasticities** |  |  |  |  |  |  |  |  |  |  |  |  |  |  |  |  |  |  |  |  |  |  |  |  |  |  |  |  |
| Millet | -1.011 | -0.019 | -0.008 | -0.004 | -0.003 | -0.017 | -0.015 | -0.009 | -0.017 | -0.005 | -0.001 | 0.000 | 0.000 | 0.000 | 0.000 | 0.000 | -0.003 | -0.025 | -0.003 | -0.006 | -0.009 | -0.001 | 0.000 | 0.000 | -0.004 | 0.000 | 0.000 | -0.002 |
| Maize | -0.003 | -1.427 | -0.009 | -0.005 | -0.004 | -0.021 | -0.017 | -0.011 | -0.020 | -0.006 | -0.001 | 0.000 | 0.000 | 0.000 | 0.000 | 0.000 | -0.004 | -0.030 | -0.004 | -0.007 | -0.011 | -0.001 | 0.000 | 0.000 | -0.005 | 0.000 | -0.001 | -0.002 |
| Rice | -0.003 | -0.019 | -0.976 | -0.004 | -0.003 | -0.017 | -0.015 | -0.009 | -0.017 | -0.005 | -0.001 | 0.000 | 0.000 | 0.000 | 0.000 | 0.000 | -0.003 | -0.025 | -0.003 | -0.006 | -0.009 | -0.001 | 0.000 | 0.000 | -0.004 | 0.000 | 0.000 | -0.002 |
| Bread | -0.003 | -0.019 | -0.008 | -1.193 | -0.003 | -0.017 | -0.015 | -0.009 | -0.017 | -0.005 | -0.001 | 0.000 | 0.000 | 0.000 | 0.000 | 0.000 | -0.003 | -0.025 | -0.003 | -0.006 | -0.009 | -0.001 | 0.000 | 0.000 | -0.004 | 0.000 | 0.000 | -0.002 |
| Sorghum | -0.003 | -0.019 | -0.008 | -0.004 | -0.992 | -0.017 | -0.015 | -0.009 | -0.017 | -0.005 | -0.001 | 0.000 | 0.000 | 0.000 | 0.000 | 0.000 | -0.003 | -0.025 | -0.003 | -0.006 | -0.009 | -0.001 | 0.000 | 0.000 | -0.004 | 0.000 | 0.000 | -0.002 |
| Sweet and Irish Potatoes | -0.004 | -0.024 | -0.010 | -0.006 | -0.004 | -0.703 | -0.019 | -0.012 | -0.021 | -0.006 | -0.001 | 0.000 | 0.000 | 0.000 | 0.000 | 0.000 | -0.004 | -0.032 | -0.004 | -0.007 | -0.012 | -0.002 | 0.000 | 0.000 | -0.005 | 0.000 | -0.001 | -0.002 |
| Cassava | -0.003 | -0.019 | -0.008 | -0.005 | -0.003 | -0.018 | -0.690 | -0.009 | -0.017 | -0.005 | -0.001 | 0.000 | 0.000 | 0.000 | 0.000 | 0.000 | -0.003 | -0.025 | -0.003 | -0.006 | -0.009 | -0.001 | 0.000 | 0.000 | -0.004 | 0.000 | -0.001 | -0.002 |
| Matooke | -0.006 | -0.039 | -0.016 | -0.009 | -0.006 | -0.037 | -0.031 | -1.263 | -0.035 | -0.010 | -0.002 | 0.000 | 0.000 | -0.001 | 0.000 | 0.000 | -0.007 | -0.052 | -0.007 | -0.012 | -0.019 | -0.002 | -0.001 | -0.001 | -0.009 | -0.001 | -0.001 | -0.004 |
| Vegetables | -0.002 | -0.013 | -0.006 | -0.003 | -0.002 | -0.012 | -0.010 | -0.007 | -0.637 | -0.003 | -0.001 | 0.000 | 0.000 | 0.000 | 0.000 | 0.000 | -0.002 | -0.018 | -0.002 | -0.004 | -0.006 | -0.001 | 0.000 | 0.000 | -0.003 | 0.000 | 0.000 | -0.001 |
| Fruits | -0.005 | -0.033 | -0.014 | -0.008 | -0.005 | -0.031 | -0.026 | -0.016 | -0.030 | -0.633 | -0.001 | 0.000 | 0.000 | -0.001 | 0.000 | 0.000 | -0.006 | -0.044 | -0.006 | -0.010 | -0.016 | -0.002 | -0.001 | -0.001 | -0.007 | -0.001 | -0.001 | -0.003 |
| Beef | -0.009 | -0.058 | -0.024 | -0.014 | -0.009 | -0.055 | -0.046 | -0.029 | -0.052 | -0.015 | -0.881 | -0.001 | -0.001 | -0.001 | 0.000 | 0.000 | -0.010 | -0.077 | -0.010 | -0.018 | -0.028 | -0.004 | -0.001 | -0.001 | -0.013 | -0.001 | -0.002 | -0.006 |
| Pork | -0.009 | -0.058 | -0.024 | -0.014 | -0.009 | -0.055 | -0.046 | -0.029 | -0.052 | -0.015 | -0.002 | -0.916 | -0.001 | -0.001 | 0.000 | 0.000 | -0.010 | -0.077 | -0.010 | -0.018 | -0.028 | -0.004 | -0.001 | -0.001 | -0.013 | -0.001 | -0.002 | -0.006 |
| Goat meat | -0.009 | -0.058 | -0.024 | -0.014 | -0.009 | -0.055 | -0.046 | -0.029 | -0.052 | -0.015 | -0.002 | -0.001 | -0.912 | -0.001 | 0.000 | 0.000 | -0.010 | -0.077 | -0.010 | -0.018 | -0.028 | -0.004 | -0.001 | -0.001 | -0.013 | -0.001 | -0.002 | -0.006 |
| Chicken | -0.009 | -0.058 | -0.024 | -0.014 | -0.009 | -0.055 | -0.046 | -0.029 | -0.052 | -0.015 | -0.002 | -0.001 | -0.001 | -0.873 | 0.000 | 0.000 | -0.010 | -0.077 | -0.010 | -0.018 | -0.028 | -0.004 | -0.001 | -0.001 | -0.013 | -0.001 | -0.002 | -0.006 |
| Other meat | -0.009 | -0.058 | -0.024 | -0.014 | -0.009 | -0.054 | -0.045 | -0.029 | -0.052 | -0.015 | -0.002 | -0.001 | -0.001 | -0.001 | -0.890 | 0.000 | -0.010 | -0.077 | -0.010 | -0.018 | -0.028 | -0.004 | -0.001 | -0.001 | -0.013 | -0.001 | -0.002 | -0.006 |
| Eggs | -0.009 | -0.059 | -0.025 | -0.014 | -0.010 | -0.055 | -0.046 | -0.029 | -0.052 | -0.015 | -0.002 | -0.001 | -0.001 | -0.001 | 0.000 | -0.994 | -0.010 | -0.078 | -0.010 | -0.018 | -0.029 | -0.004 | -0.001 | -0.001 | -0.013 | -0.001 | -0.002 | -0.006 |
| Fish | -0.007 | -0.047 | -0.020 | -0.011 | -0.008 | -0.044 | -0.037 | -0.023 | -0.042 | -0.012 | -0.002 | 0.000 | 0.000 | -0.001 | 0.000 | 0.000 | -1.330 | -0.063 | -0.008 | -0.014 | -0.023 | -0.003 | -0.001 | -0.001 | -0.011 | -0.001 | -0.001 | -0.004 |
| Pulses, legumes, nuts | -0.003 | -0.019 | -0.008 | -0.005 | -0.003 | -0.018 | -0.015 | -0.009 | -0.017 | -0.005 | -0.001 | 0.000 | 0.000 | 0.000 | 0.000 | 0.000 | -0.003 | -0.837 | -0.003 | -0.006 | -0.009 | -0.001 | 0.000 | 0.000 | -0.004 | 0.000 | -0.001 | -0.002 |
| Milk | -0.007 | -0.043 | -0.018 | -0.010 | -0.007 | -0.040 | -0.034 | -0.021 | -0.038 | -0.011 | -0.002 | 0.000 | 0.000 | -0.001 | 0.000 | 0.000 | -0.007 | -0.057 | -1.400 | -0.013 | -0.021 | -0.003 | -0.001 | -0.001 | -0.010 | -0.001 | -0.001 | -0.004 |
| Oils and fats | -0.003 | -0.022 | -0.009 | -0.005 | -0.004 | -0.021 | -0.017 | -0.011 | -0.020 | -0.006 | -0.001 | 0.000 | 0.000 | 0.000 | 0.000 | 0.000 | -0.004 | -0.030 | -0.004 | -0.673 | -0.011 | -0.001 | 0.000 | 0.000 | -0.005 | 0.000 | -0.001 | -0.002 |
| Sugar | -0.004 | -0.023 | -0.010 | -0.006 | -0.004 | -0.022 | -0.018 | -0.011 | -0.021 | -0.006 | -0.001 | 0.000 | 0.000 | 0.000 | 0.000 | 0.000 | -0.004 | -0.031 | -0.004 | -0.007 | -1.038 | -0.001 | 0.000 | 0.000 | -0.005 | 0.000 | -0.001 | -0.002 |
| Soda | -0.004 | -0.023 | -0.010 | -0.006 | -0.004 | -0.022 | -0.018 | -0.011 | -0.021 | -0.006 | -0.001 | 0.000 | 0.000 | 0.000 | 0.000 | 0.000 | -0.004 | -0.031 | -0.004 | -0.007 | -0.011 | -0.973 | 0.000 | 0.000 | -0.005 | 0.000 | -0.001 | -0.002 |
| Other juices | -0.004 | -0.023 | -0.010 | -0.006 | -0.004 | -0.022 | -0.018 | -0.011 | -0.021 | -0.006 | -0.001 | 0.000 | 0.000 | 0.000 | 0.000 | 0.000 | -0.004 | -0.031 | -0.004 | -0.007 | -0.011 | -0.001 | -0.982 | 0.000 | -0.005 | 0.000 | -0.001 | -0.002 |
| Coffee & tea | -0.006 | -0.042 | -0.018 | -0.010 | -0.007 | -0.040 | -0.033 | -0.021 | -0.038 | -0.011 | -0.002 | 0.000 | 0.000 | -0.001 | 0.000 | 0.000 | -0.007 | -0.056 | -0.007 | -0.013 | -0.021 | -0.003 | -0.001 | -0.927 | -0.009 | -0.001 | -0.001 | -0.004 |
| Food away | -0.006 | -0.043 | -0.018 | -0.010 | -0.007 | -0.040 | -0.033 | -0.021 | -0.038 | -0.011 | -0.002 | 0.000 | 0.000 | -0.001 | 0.000 | 0.000 | -0.007 | -0.056 | -0.007 | -0.013 | -0.021 | -0.003 | -0.001 | -0.001 | -0.887 | -0.001 | -0.001 | -0.004 |
| Beer | -0.006 | -0.042 | -0.018 | -0.010 | -0.007 | -0.040 | -0.033 | -0.021 | -0.038 | -0.011 | -0.002 | 0.000 | 0.000 | -0.001 | 0.000 | 0.000 | -0.007 | -0.056 | -0.007 | -0.013 | -0.021 | -0.003 | -0.001 | -0.001 | -0.009 | -0.733 | -0.001 | -0.004 |
| Other alcoholic beverages | -0.006 | -0.043 | -0.018 | -0.010 | -0.007 | -0.040 | -0.033 | -0.021 | -0.038 | -0.011 | -0.002 | 0.000 | 0.000 | -0.001 | 0.000 | 0.000 | -0.007 | -0.056 | -0.007 | -0.013 | -0.021 | -0.003 | -0.001 | -0.001 | -0.009 | -0.001 | -2.654 | -0.004 |
| Other foods | -0.006 | -0.042 | -0.018 | -0.010 | -0.007 | -0.040 | -0.033 | -0.021 | -0.038 | -0.011 | -0.002 | 0.000 | 0.000 | -0.001 | 0.000 | 0.000 | -0.007 | -0.056 | -0.007 | -0.013 | -0.021 | -0.003 | -0.001 | -0.001 | -0.009 | -0.001 | -0.001 | -0.946 |
| **Income elasticities** | 0.596 | 0.713 | 0.596 | 0.598 | 0.596 | 0.773 | 0.613 | 1.263 | 0.427 | 1.073 | 1.869 | 1.870 | 1.871 | 1.870 | 1.865 | 1.880 | 1.513 | 0.617 | 1.383 | 0.717 | 0.747 | 0.746 | 0.747 | 1.360 | 1.364 | 1.359 | 1.365 | 1.360 |
|  |  |  |  |  |  |  |  |  |  |  |  |  |  |  |  |  |  |  |  |  |  |  |  |  |  |  |  |  |

Table A3: Calibrated elasticities - Group urban-upper quintiles

|  | Millet | Maize | Rice | Bread | Sorgh. | Sweet | Cass. | Matoo. | Veget. | Fruits | Beef | Pork | Goat | Chick. | Other | Eggs | Fish | Puls., | Milk | Oils | Sugar | Soda | Other | Coffee | Food | Beer | Other | Other |
| --- | --- | --- | --- | --- | --- | --- | --- | --- | --- | --- | --- | --- | --- | --- | --- | --- | --- | --- | --- | --- | --- | --- | --- | --- | --- | --- | --- | --- |
|  |  |  |  |  |  | Irish |  |  |  |  |  |  | meat |  | meat |  |  | leg., |  | and |  |  | juices | and | away |  | alcoh. | foods |
|  |  |  |  |  |  | Potat. |  |  |  |  |  |  |  |  |  |  |  | nuts |  | fats |  |  |  | tea | home |  | bev. |  |
|  |  |  |  |  |  |  |  |  |  |  |  |  |  |  |  |  |  |  |  |  |  |  |  |  |  |  |  |  |
| **Hicksian elasticities** |  |  |  |  |  |  |  |  |  |  |  |  |  |  |  |  |  |  |  |  |  |  |  |  |  |  |  |  |
| Millet | -1.013 | 0.002 | 0.002 | 0.002 | 0.000 | 0.002 | 0.001 | 0.006 | 0.001 | 0.004 | 0.007 | 0.001 | 0.001 | 0.003 | 0.000 | 0.001 | 0.004 | 0.002 | 0.003 | 0.001 | 0.002 | 0.001 | 0.000 | 0.000 | 0.016 | 0.002 | 0.000 | 0.002 |
| Maize | 0.000 | -1.724 | 0.002 | 0.002 | 0.000 | 0.002 | 0.001 | 0.007 | 0.001 | 0.004 | 0.008 | 0.001 | 0.001 | 0.003 | 0.000 | 0.001 | 0.004 | 0.003 | 0.004 | 0.001 | 0.003 | 0.001 | 0.000 | 0.000 | 0.017 | 0.002 | 0.000 | 0.002 |
| Rice | 0.000 | 0.002 | -0.971 | 0.002 | 0.000 | 0.002 | 0.001 | 0.006 | 0.001 | 0.004 | 0.007 | 0.001 | 0.001 | 0.003 | 0.000 | 0.001 | 0.004 | 0.002 | 0.003 | 0.001 | 0.002 | 0.001 | 0.000 | 0.000 | 0.016 | 0.002 | 0.000 | 0.002 |
| Bread | 0.000 | 0.002 | 0.002 | -1.338 | 0.000 | 0.002 | 0.001 | 0.006 | 0.001 | 0.004 | 0.007 | 0.001 | 0.001 | 0.003 | 0.000 | 0.001 | 0.004 | 0.002 | 0.003 | 0.001 | 0.002 | 0.001 | 0.000 | 0.000 | 0.017 | 0.002 | 0.000 | 0.002 |
| Sorghum | 0.000 | 0.002 | 0.002 | 0.002 | -1.013 | 0.002 | 0.001 | 0.006 | 0.001 | 0.004 | 0.007 | 0.001 | 0.001 | 0.003 | 0.000 | 0.001 | 0.004 | 0.002 | 0.003 | 0.001 | 0.002 | 0.001 | 0.000 | 0.000 | 0.016 | 0.002 | 0.000 | 0.002 |
| Sweet and Irish Potatoes | 0.000 | 0.002 | 0.002 | 0.002 | 0.000 | -0.398 | 0.001 | 0.008 | 0.001 | 0.004 | 0.009 | 0.001 | 0.002 | 0.003 | 0.000 | 0.002 | 0.005 | 0.003 | 0.004 | 0.001 | 0.003 | 0.001 | 0.000 | 0.000 | 0.020 | 0.002 | 0.000 | 0.002 |
| Cassava | 0.000 | 0.002 | 0.002 | 0.002 | 0.000 | 0.002 | -0.206 | 0.008 | 0.001 | 0.004 | 0.009 | 0.001 | 0.002 | 0.003 | 0.000 | 0.001 | 0.005 | 0.003 | 0.004 | 0.001 | 0.003 | 0.001 | 0.000 | 0.000 | 0.020 | 0.002 | 0.000 | 0.002 |
| Matooke | 0.001 | 0.003 | 0.003 | 0.003 | 0.000 | 0.004 | 0.002 | -0.883 | 0.002 | 0.006 | 0.013 | 0.002 | 0.002 | 0.005 | 0.000 | 0.002 | 0.007 | 0.004 | 0.006 | 0.002 | 0.004 | 0.002 | 0.000 | 0.001 | 0.029 | 0.003 | 0.001 | 0.003 |
| Vegetables | 0.000 | 0.001 | 0.001 | 0.001 | 0.000 | 0.001 | 0.001 | 0.004 | -0.467 | 0.002 | 0.004 | 0.001 | 0.001 | 0.002 | 0.000 | 0.001 | 0.002 | 0.001 | 0.002 | 0.001 | 0.001 | 0.001 | 0.000 | 0.000 | 0.009 | 0.001 | 0.000 | 0.001 |
| Fruits | 0.001 | 0.004 | 0.004 | 0.004 | 0.000 | 0.004 | 0.002 | 0.013 | 0.002 | -0.607 | 0.015 | 0.002 | 0.003 | 0.006 | 0.000 | 0.003 | 0.009 | 0.005 | 0.007 | 0.002 | 0.005 | 0.002 | 0.000 | 0.001 | 0.034 | 0.003 | 0.001 | 0.003 |
| Beef | 0.001 | 0.005 | 0.005 | 0.005 | 0.000 | 0.005 | 0.003 | 0.017 | 0.003 | 0.010 | -0.427 | 0.002 | 0.004 | 0.007 | 0.001 | 0.003 | 0.011 | 0.006 | 0.009 | 0.002 | 0.006 | 0.003 | 0.001 | 0.001 | 0.044 | 0.004 | 0.001 | 0.004 |
| Pork | 0.001 | 0.005 | 0.005 | 0.005 | 0.000 | 0.005 | 0.003 | 0.017 | 0.003 | 0.010 | 0.019 | -0.905 | 0.004 | 0.007 | 0.001 | 0.003 | 0.011 | 0.006 | 0.009 | 0.002 | 0.006 | 0.003 | 0.001 | 0.001 | 0.044 | 0.004 | 0.001 | 0.004 |
| Goat meat | 0.001 | 0.005 | 0.005 | 0.005 | 0.000 | 0.005 | 0.003 | 0.017 | 0.003 | 0.010 | 0.019 | 0.002 | -0.888 | 0.007 | 0.001 | 0.003 | 0.011 | 0.006 | 0.009 | 0.002 | 0.006 | 0.003 | 0.001 | 0.001 | 0.044 | 0.004 | 0.001 | 0.004 |
| Chicken | 0.001 | 0.005 | 0.005 | 0.005 | 0.000 | 0.005 | 0.003 | 0.017 | 0.003 | 0.010 | 0.019 | 0.002 | 0.004 | -0.814 | 0.001 | 0.003 | 0.011 | 0.006 | 0.009 | 0.002 | 0.006 | 0.003 | 0.001 | 0.001 | 0.044 | 0.004 | 0.001 | 0.004 |
| Other meat | 0.001 | 0.005 | 0.005 | 0.005 | 0.000 | 0.005 | 0.003 | 0.017 | 0.003 | 0.010 | 0.019 | 0.002 | 0.004 | 0.007 | -0.343 | 0.003 | 0.011 | 0.006 | 0.009 | 0.002 | 0.006 | 0.003 | 0.001 | 0.001 | 0.044 | 0.004 | 0.001 | 0.004 |
| Eggs | 0.001 | 0.005 | 0.005 | 0.005 | 0.000 | 0.005 | 0.003 | 0.017 | 0.003 | 0.010 | 0.019 | 0.002 | 0.004 | 0.007 | 0.001 | -0.944 | 0.011 | 0.006 | 0.009 | 0.002 | 0.006 | 0.003 | 0.001 | 0.001 | 0.044 | 0.004 | 0.001 | 0.004 |
| Fish | 0.001 | 0.005 | 0.004 | 0.004 | 0.000 | 0.005 | 0.003 | 0.016 | 0.002 | 0.009 | 0.019 | 0.002 | 0.003 | 0.007 | 0.001 | 0.003 | -1.235 | 0.006 | 0.009 | 0.002 | 0.006 | 0.003 | 0.001 | 0.001 | 0.043 | 0.004 | 0.001 | 0.004 |
| Pulses, legumes, nuts | 0.000 | 0.002 | 0.002 | 0.002 | 0.000 | 0.002 | 0.001 | 0.006 | 0.001 | 0.003 | 0.006 | 0.001 | 0.001 | 0.002 | 0.000 | 0.001 | 0.004 | -0.757 | 0.003 | 0.001 | 0.002 | 0.001 | 0.000 | 0.000 | 0.015 | 0.001 | 0.000 | 0.001 |
| Milk | 0.001 | 0.003 | 0.003 | 0.003 | 0.000 | 0.004 | 0.002 | 0.012 | 0.002 | 0.007 | 0.014 | 0.002 | 0.003 | 0.005 | 0.000 | 0.002 | 0.008 | 0.004 | -1.168 | 0.002 | 0.004 | 0.002 | 0.000 | 0.001 | 0.031 | 0.003 | 0.001 | 0.003 |
| Oils and fats | 0.000 | 0.002 | 0.002 | 0.002 | 0.000 | 0.002 | 0.001 | 0.006 | 0.001 | 0.003 | 0.007 | 0.001 | 0.001 | 0.003 | 0.000 | 0.001 | 0.004 | 0.002 | 0.003 | -0.557 | 0.002 | 0.001 | 0.000 | 0.000 | 0.015 | 0.001 | 0.000 | 0.001 |
| Sugar | 0.000 | 0.002 | 0.002 | 0.002 | 0.000 | 0.003 | 0.001 | 0.009 | 0.001 | 0.005 | 0.010 | 0.001 | 0.002 | 0.004 | 0.000 | 0.002 | 0.006 | 0.003 | 0.005 | 0.001 | -0.966 | 0.001 | 0.000 | 0.000 | 0.022 | 0.002 | 0.000 | 0.002 |
| Soda | 0.000 | 0.002 | 0.002 | 0.002 | 0.000 | 0.003 | 0.001 | 0.009 | 0.001 | 0.005 | 0.010 | 0.001 | 0.002 | 0.004 | 0.000 | 0.002 | 0.006 | 0.003 | 0.005 | 0.001 | 0.003 | -0.974 | 0.000 | 0.000 | 0.022 | 0.002 | 0.000 | 0.002 |
| Other juices | 0.000 | 0.002 | 0.002 | 0.002 | 0.000 | 0.003 | 0.001 | 0.009 | 0.001 | 0.005 | 0.010 | 0.001 | 0.002 | 0.004 | 0.000 | 0.002 | 0.006 | 0.003 | 0.005 | 0.001 | 0.003 | 0.001 | -0.987 | 0.000 | 0.022 | 0.002 | 0.000 | 0.002 |
| Coffee & tea | 0.001 | 0.005 | 0.005 | 0.005 | 0.000 | 0.006 | 0.003 | 0.017 | 0.003 | 0.010 | 0.020 | 0.002 | 0.004 | 0.008 | 0.001 | 0.003 | 0.011 | 0.006 | 0.009 | 0.002 | 0.007 | 0.003 | 0.001 | -0.844 | 0.045 | 0.004 | 0.001 | 0.004 |
| Food away | 0.001 | 0.005 | 0.005 | 0.005 | 0.000 | 0.006 | 0.003 | 0.017 | 0.003 | 0.010 | 0.020 | 0.002 | 0.004 | 0.008 | 0.001 | 0.003 | 0.011 | 0.006 | 0.009 | 0.002 | 0.007 | 0.003 | 0.001 | 0.001 | -0.840 | 0.004 | 0.001 | 0.004 |
| Beer | 0.001 | 0.005 | 0.005 | 0.005 | 0.000 | 0.006 | 0.003 | 0.017 | 0.003 | 0.010 | 0.020 | 0.002 | 0.004 | 0.008 | 0.001 | 0.003 | 0.011 | 0.006 | 0.009 | 0.002 | 0.007 | 0.003 | 0.001 | 0.001 | 0.045 | -0.286 | 0.001 | 0.004 |
| Other alcoholic beverages | 0.001 | 0.005 | 0.005 | 0.005 | 0.000 | 0.006 | 0.003 | 0.017 | 0.003 | 0.010 | 0.020 | 0.003 | 0.004 | 0.008 | 0.001 | 0.003 | 0.011 | 0.006 | 0.009 | 0.002 | 0.007 | 0.003 | 0.001 | 0.001 | 0.045 | 0.004 | -7.492 | 0.004 |
| Other foods | 0.001 | 0.005 | 0.005 | 0.005 | 0.000 | 0.006 | 0.003 | 0.017 | 0.003 | 0.010 | 0.020 | 0.002 | 0.004 | 0.008 | 0.001 | 0.003 | 0.011 | 0.006 | 0.009 | 0.002 | 0.006 | 0.003 | 0.001 | 0.001 | 0.045 | 0.004 | 0.001 | -1.000 |
| **Marshallian elasticities** |  |  |  |  |  |  |  |  |  |  |  |  |  |  |  |  |  |  |  |  |  |  |  |  |  |  |  |  |
| Millet | -1.014 | -0.004 | -0.004 | -0.004 | 0.000 | -0.004 | -0.002 | -0.006 | -0.005 | -0.003 | -0.002 | 0.000 | 0.000 | -0.001 | 0.000 | 0.000 | -0.002 | -0.007 | -0.003 | -0.003 | -0.004 | -0.002 | 0.000 | 0.000 | -0.005 | 0.000 | 0.000 | 0.000 |
| Maize | -0.001 | -1.730 | -0.005 | -0.005 | 0.000 | -0.004 | -0.002 | -0.007 | -0.006 | -0.003 | -0.003 | 0.000 | 0.000 | -0.001 | 0.000 | 0.000 | -0.002 | -0.008 | -0.003 | -0.003 | -0.004 | -0.002 | 0.000 | 0.000 | -0.005 | 0.000 | 0.000 | -0.001 |
| Rice | -0.001 | -0.004 | -0.977 | -0.004 | 0.000 | -0.004 | -0.002 | -0.006 | -0.005 | -0.003 | -0.002 | 0.000 | 0.000 | -0.001 | 0.000 | 0.000 | -0.002 | -0.007 | -0.003 | -0.003 | -0.004 | -0.002 | 0.000 | 0.000 | -0.005 | 0.000 | 0.000 | 0.000 |
| Bread | -0.001 | -0.004 | -0.004 | -1.344 | 0.000 | -0.004 | -0.002 | -0.006 | -0.005 | -0.003 | -0.002 | 0.000 | 0.000 | -0.001 | 0.000 | 0.000 | -0.002 | -0.007 | -0.003 | -0.003 | -0.004 | -0.002 | 0.000 | 0.000 | -0.005 | 0.000 | 0.000 | 0.000 |
| Sorghum | -0.001 | -0.004 | -0.004 | -0.004 | -1.013 | -0.004 | -0.002 | -0.006 | -0.005 | -0.003 | -0.002 | 0.000 | 0.000 | -0.001 | 0.000 | 0.000 | -0.002 | -0.007 | -0.003 | -0.003 | -0.004 | -0.002 | 0.000 | 0.000 | -0.005 | 0.000 | 0.000 | 0.000 |
| Sweet and Irish Potatoes | -0.001 | -0.005 | -0.005 | -0.005 | 0.000 | -0.405 | -0.003 | -0.008 | -0.006 | -0.003 | -0.003 | 0.000 | -0.001 | -0.001 | 0.000 | -0.001 | -0.002 | -0.009 | -0.004 | -0.003 | -0.005 | -0.002 | 0.000 | 0.000 | -0.006 | -0.001 | 0.000 | -0.001 |
| Cassava | -0.001 | -0.005 | -0.005 | -0.005 | 0.000 | -0.005 | -0.210 | -0.008 | -0.006 | -0.003 | -0.003 | 0.000 | -0.001 | -0.001 | 0.000 | 0.000 | -0.002 | -0.009 | -0.004 | -0.003 | -0.005 | -0.002 | 0.000 | 0.000 | -0.006 | -0.001 | 0.000 | -0.001 |
| Matooke | -0.002 | -0.007 | -0.008 | -0.008 | 0.000 | -0.007 | -0.004 | -0.905 | -0.009 | -0.004 | -0.004 | -0.001 | -0.001 | -0.002 | 0.000 | -0.001 | -0.003 | -0.013 | -0.005 | -0.005 | -0.007 | -0.003 | -0.001 | 0.000 | -0.009 | -0.001 | 0.000 | -0.001 |
| Vegetables | 0.000 | -0.002 | -0.002 | -0.002 | 0.000 | -0.002 | -0.001 | -0.004 | -0.470 | -0.001 | -0.001 | 0.000 | 0.000 | -0.001 | 0.000 | 0.000 | -0.001 | -0.004 | -0.002 | -0.001 | -0.002 | -0.001 | 0.000 | 0.000 | -0.003 | 0.000 | 0.000 | 0.000 |
| Fruits | -0.002 | -0.009 | -0.009 | -0.009 | 0.000 | -0.008 | -0.004 | -0.013 | -0.011 | -0.620 | -0.005 | -0.001 | -0.001 | -0.002 | 0.000 | -0.001 | -0.003 | -0.015 | -0.006 | -0.005 | -0.008 | -0.004 | -0.001 | 0.000 | -0.010 | -0.001 | 0.000 | -0.001 |
| Beef | -0.002 | -0.011 | -0.012 | -0.012 | 0.000 | -0.010 | -0.006 | -0.017 | -0.014 | -0.007 | -0.453 | -0.001 | -0.001 | -0.003 | 0.000 | -0.001 | -0.004 | -0.019 | -0.008 | -0.007 | -0.010 | -0.005 | -0.001 | 0.000 | -0.013 | -0.001 | 0.000 | -0.001 |
| Pork | -0.002 | -0.011 | -0.012 | -0.012 | 0.000 | -0.010 | -0.006 | -0.017 | -0.014 | -0.007 | -0.007 | -0.908 | -0.001 | -0.003 | 0.000 | -0.001 | -0.004 | -0.019 | -0.008 | -0.007 | -0.010 | -0.005 | -0.001 | 0.000 | -0.013 | -0.001 | 0.000 | -0.001 |
| Goat meat | -0.002 | -0.011 | -0.012 | -0.012 | 0.000 | -0.010 | -0.006 | -0.017 | -0.014 | -0.007 | -0.007 | -0.001 | -0.893 | -0.003 | 0.000 | -0.001 | -0.004 | -0.019 | -0.008 | -0.007 | -0.010 | -0.005 | -0.001 | 0.000 | -0.013 | -0.001 | 0.000 | -0.001 |
| Chicken | -0.002 | -0.011 | -0.012 | -0.012 | 0.000 | -0.010 | -0.006 | -0.017 | -0.014 | -0.007 | -0.007 | -0.001 | -0.001 | -0.824 | 0.000 | -0.001 | -0.004 | -0.019 | -0.008 | -0.007 | -0.010 | -0.005 | -0.001 | 0.000 | -0.013 | -0.001 | 0.000 | -0.001 |
| Other meat | -0.002 | -0.011 | -0.012 | -0.012 | 0.000 | -0.010 | -0.006 | -0.017 | -0.014 | -0.007 | -0.007 | -0.001 | -0.001 | -0.002 | -0.344 | -0.001 | -0.004 | -0.019 | -0.008 | -0.007 | -0.010 | -0.005 | -0.001 | 0.000 | -0.013 | -0.001 | 0.000 | -0.001 |
| Eggs | -0.002 | -0.011 | -0.012 | -0.012 | 0.000 | -0.010 | -0.006 | -0.017 | -0.014 | -0.007 | -0.007 | -0.001 | -0.001 | -0.003 | 0.000 | -0.949 | -0.004 | -0.019 | -0.008 | -0.007 | -0.010 | -0.005 | -0.001 | 0.000 | -0.013 | -0.001 | 0.000 | -0.001 |
| Fish | -0.002 | -0.011 | -0.011 | -0.011 | 0.000 | -0.010 | -0.005 | -0.017 | -0.013 | -0.007 | -0.006 | -0.001 | -0.001 | -0.002 | 0.000 | -0.001 | -1.250 | -0.018 | -0.008 | -0.007 | -0.010 | -0.005 | -0.001 | 0.000 | -0.013 | -0.001 | 0.000 | -0.001 |
| Pulses, legumes, nuts | -0.001 | -0.004 | -0.004 | -0.004 | 0.000 | -0.003 | -0.002 | -0.006 | -0.005 | -0.002 | -0.002 | 0.000 | 0.000 | -0.001 | 0.000 | 0.000 | -0.001 | -0.765 | -0.003 | -0.002 | -0.003 | -0.002 | 0.000 | 0.000 | -0.004 | 0.000 | 0.000 | 0.000 |
| Milk | -0.002 | -0.008 | -0.008 | -0.008 | 0.000 | -0.007 | -0.004 | -0.012 | -0.010 | -0.005 | -0.005 | -0.001 | -0.001 | -0.002 | 0.000 | -0.001 | -0.003 | -0.013 | -1.180 | -0.005 | -0.007 | -0.003 | -0.001 | 0.000 | -0.009 | -0.001 | 0.000 | -0.001 |
| Oils and fats | -0.001 | -0.004 | -0.004 | -0.004 | 0.000 | -0.004 | -0.002 | -0.006 | -0.005 | -0.002 | -0.002 | 0.000 | 0.000 | -0.001 | 0.000 | 0.000 | -0.001 | -0.007 | -0.003 | -0.560 | -0.004 | -0.002 | 0.000 | 0.000 | -0.005 | 0.000 | 0.000 | 0.000 |
| Sugar | -0.001 | -0.006 | -0.006 | -0.006 | 0.000 | -0.005 | -0.003 | -0.009 | -0.007 | -0.003 | -0.003 | 0.000 | -0.001 | -0.001 | 0.000 | -0.001 | -0.002 | -0.010 | -0.004 | -0.003 | -0.975 | -0.002 | 0.000 | 0.000 | -0.007 | -0.001 | 0.000 | -0.001 |
| Soda | -0.001 | -0.006 | -0.006 | -0.006 | 0.000 | -0.005 | -0.003 | -0.009 | -0.007 | -0.003 | -0.003 | 0.000 | -0.001 | -0.001 | 0.000 | -0.001 | -0.002 | -0.010 | -0.004 | -0.003 | -0.005 | -0.977 | 0.000 | 0.000 | -0.007 | -0.001 | 0.000 | -0.001 |
| Other juices | -0.001 | -0.006 | -0.006 | -0.006 | 0.000 | -0.005 | -0.003 | -0.009 | -0.007 | -0.003 | -0.003 | 0.000 | -0.001 | -0.001 | 0.000 | -0.001 | -0.002 | -0.010 | -0.004 | -0.003 | -0.005 | -0.002 | -0.988 | 0.000 | -0.007 | -0.001 | 0.000 | -0.001 |
| Coffee & tea | -0.002 | -0.011 | -0.012 | -0.012 | 0.000 | -0.011 | -0.006 | -0.017 | -0.014 | -0.007 | -0.007 | -0.001 | -0.001 | -0.003 | 0.000 | -0.001 | -0.004 | -0.019 | -0.008 | -0.007 | -0.011 | -0.005 | -0.001 | -0.845 | -0.014 | -0.001 | 0.000 | -0.001 |
| Food away | -0.002 | -0.011 | -0.012 | -0.012 | 0.000 | -0.011 | -0.006 | -0.018 | -0.014 | -0.007 | -0.007 | -0.001 | -0.001 | -0.003 | 0.000 | -0.001 | -0.004 | -0.019 | -0.008 | -0.007 | -0.011 | -0.005 | -0.001 | 0.000 | -0.899 | -0.001 | 0.000 | -0.001 |
| Beer | -0.002 | -0.011 | -0.012 | -0.012 | 0.000 | -0.011 | -0.006 | -0.017 | -0.014 | -0.007 | -0.007 | -0.001 | -0.001 | -0.003 | 0.000 | -0.001 | -0.004 | -0.019 | -0.008 | -0.007 | -0.011 | -0.005 | -0.001 | 0.000 | -0.014 | -0.291 | 0.000 | -0.001 |
| Other alcoholic beverages | -0.002 | -0.011 | -0.012 | -0.012 | 0.000 | -0.011 | -0.006 | -0.018 | -0.014 | -0.007 | -0.007 | -0.001 | -0.001 | -0.003 | 0.000 | -0.001 | -0.004 | -0.019 | -0.008 | -0.007 | -0.011 | -0.005 | -0.001 | 0.000 | -0.014 | -0.001 | -7.493 | -0.001 |
| Other foods | -0.002 | -0.011 | -0.012 | -0.012 | 0.000 | -0.010 | -0.006 | -0.017 | -0.014 | -0.007 | -0.007 | -0.001 | -0.001 | -0.003 | 0.000 | -0.001 | -0.004 | -0.019 | -0.008 | -0.007 | -0.011 | -0.005 | -0.001 | 0.000 | -0.014 | -0.001 | 0.000 | -1.005 |
| **Income elasticities** | 0.405 | 0.430 | 0.405 | 0.406 | 0.404 | 0.500 | 0.485 | 0.715 | 0.225 | 0.845 | 1.074 | 1.075 | 1.075 | 1.075 | 1.072 | 1.079 | 1.045 | 0.360 | 0.755 | 0.375 | 0.545 | 0.545 | 0.545 | 1.099 | 1.106 | 1.103 | 1.110 | 1.097 |
|  |  |  |  |  |  |  |  |  |  |  |  |  |  |  |  |  |  |  |  |  |  |  |  |  |  |  |  |  |

Table A4: Food composition (based on 100 grams)

|  | **Nutrients** | | | | | | | | | | | | | |
| --- | --- | --- | --- | --- | --- | --- | --- | --- | --- | --- | --- | --- | --- | --- |
|  | Energy | Protein | Lipid total | Carbohydrate | Calcium | Iron | Zinc | Vitamin C | Thiamin | Riboflavin | Niacin | Vitamin B6 | Folate | Vitamin A |
|  | (kcal) | (g) | (g) | (g) | (g) | (mg) | (mg) | (mg) | Vitamin B1 | Vitamin B2 | Vitamin B3 | (mg) | (μg dietary | (μg retinol |
|  |  |  |  |  |  |  |  |  | (mg) | (mg) | (mg) |  | folate | equivalent) |
|  |  |  |  |  |  |  |  |  |  |  |  |  | equivalence) |  |
|  |  |  |  |  |  |  |  |  |  |  |  |  |  |  |
| Millet flour | 374.000 | 10.900 | 4.200 | 72.100 | 8.000 | 3.000 | 1.700 | 0.000 | 0.416 | 0.287 | 4.668 | 0.380 | 84.000 | 0.000 |
| Maize | 369.000 | 7.300 | 1.800 | 79.200 | 3.000 | 1.100 | 0.700 | 0.000 | 0.140 | 0.050 | 1.000 | 0.198 | 30.000 | 0.000 |
| Rice | 361.000 | 7.050 | 1.650 | 77.750 | 21.000 | 1.300 | 1.600 | 0.000 | 0.242 | 0.046 | 2.954 | 0.327 | 14.500 | 0.000 |
| Bread | 266.000 | 7.600 | 3.300 | 50.600 | 151.000 | 3.700 | 0.700 | 0.000 | 0.455 | 0.331 | 4.385 | 0.084 | 25.000 | 0.000 |
| Sorghum | 339.000 | 11.300 | 3.300 | 74.600 | 28.000 | 4.400 | 1.600 | 0.000 | 0.237 | 0.142 | 2.927 | 0.150 | 14.000 | 0.000 |
| Sweet and Irish Potatoes | 97.000 | 2.100 | 0.100 | 22.450 | 26.500 | 0.800 | 0.350 | 11.500 | 0.094 | 0.058 | 0.908 | 0.290 | 15.500 | 0.000 |
| Cassava | 237.000 | 2.000 | 0.500 | 57.350 | 23.500 | 1.100 | 0.500 | 46.300 | 0.199 | 0.049 | 1.127 | 0.394 | 31.500 | 4.000 |
| Matooke | 122.000 | 1.300 | 0.400 | 31.900 | 3.000 | 0.600 | 0.100 | 18.400 | 0.052 | 0.054 | 0.686 | 0.299 | 22.000 | 56.000 |
| Vegetables | 31.500 | 1.150 | 0.150 | 7.200 | 18.000 | 0.350 | 0.150 | 15.400 | 0.053 | 0.034 | 0.308 | 0.101 | 14.000 | 16.000 |
| Fruits | 72.000 | 0.800 | 0.200 | 18.400 | 6.000 | 0.850 | 1.850 | 47.350 | 0.026 | 0.052 | 0.433 | 0.234 | 16.000 | 4.000 |
| Beef | 251.000 | 18.200 | 19.200 | 0.000 | 7.000 | 1.900 | 3.700 | 0.000 | 0.090 | 0.160 | 3.150 | 0.380 | 6.000 | 0.000 |
| Pork | 200.000 | 19.500 | 12.900 | 0.000 | 19.000 | 0.800 | 1.900 | 0.600 | 0.892 | 0.253 | 4.492 | 0.456 | 5.000 | 2.000 |
| Goat meat | 109.000 | 20.600 | 2.300 | 0.000 | 13.000 | 2.800 | 4.000 | 0.000 | 0.110 | 0.490 | 3.750 | 0.200 | 5.000 | 0.000 |
| Chicken | 215.000 | 18.600 | 15.100 | 0.000 | 11.000 | 0.900 | 1.300 | 1.600 | 0.060 | 0.120 | 6.801 | 0.350 | 6.000 | 42.000 |
| Other meat | 222.000 | 18.300 | 16.000 | 0.000 | 12.000 | 1.000 | 1.300 | 0.700 | 0.048 | 0.088 | 5.926 | 0.350 | 4.000 | 44.000 |
| Eggs | 143.000 | 12.600 | 9.900 | 0.800 | 53.000 | 1.800 | 1.100 | 0.000 | 0.069 | 0.478 | 0.070 | 0.143 | 47.000 | 140.000 |
| Fish | 96.000 | 20.100 | 1.700 | 0.000 | 10.000 | 0.600 | 0.300 | 0.000 | 0.041 | 0.063 | 3.903 | 0.162 | 24.000 | 0.000 |
| Pulses, legumes, nuts | 457.000 | 23.600 | 25.200 | 39.350 | 102.500 | 4.850 | 2.800 | 3.150 | 0.677 | 0.174 | 6.620 | 0.411 | 382.500 | 0.000 |
| Milk | 60.000 | 3.200 | 3.300 | 4.500 | 113.000 | 0.000 | 0.400 | 0.000 | 0.044 | 0.183 | 0.107 | 0.036 | 5.000 | 28.000 |
| Oils and fats | 884.000 | 0.000 | 100.000 | 0.000 | 0.000 | 0.000 | 0.000 | 0.000 | 0.000 | 0.000 | 0.000 | 0.000 | 0.000 | 0.000 |
| Sugar | 54.000 | 0.600 | 0.100 | 13.000 | 8.000 | 1.400 | 0.000 | 3.000 | 0.020 | 0.010 | 0.100 | 0.000 | 0.000 | 0.000 |
| Soda | 48.000 | 0.000 | 0.000 | 12.300 | 5.000 | 0.100 | 0.100 | 0.000 | 0.000 | 0.000 | 0.000 | 0.000 | 0.000 | 0.000 |
| Other juices | 45.000 | 0.700 | 0.200 | 10.400 | 11.000 | 0.200 | 0.050 | 50.000 | 0.090 | 0.030 | 0.400 | 0.040 | 30.000 | 10.000 |
| Coffee & tea | 1.000 | 0.100 | 0.000 | 0.200 | 0.000 | 0.010 | 0.000 | 0.000 | 1.000 | 0.000 | 1.000 | 2.000 | 0.000 | 0.000 |
| Beer | 41.000 | 0.300 | 0.000 | 3.700 | 5.000 | 0.000 | 0.000 | 0.000 | 0.010 | 0.030 | 0.510 | 0.050 | 6.000 | 0.000 |
| Other alcoholic beverages | 263.000 | 0.000 | 0.000 | 0.000 | 0.000 | 0.000 | 0.000 | 0.000 | 0.000 | 0.000 | 0.000 | 0.000 | 0.000 | 0.000 |
|  |  |  |  |  |  |  |  |  |  |  |  |  |  |  |

Source: Hotz et al. (2012).
